# Highly Efficient Modular Construction of Functional Drug Delivery Platform Based on Amphiphilic Biodegradable Polymers via Click Chemistry

**DOI:** 10.3390/ijms221910407

**Published:** 2021-09-27

**Authors:** Guangkuo Zhao, Tongtong Ge, Yunfeng Yan, Qi Shuai, Wei-Ke Su

**Affiliations:** 1 Collaborative Innovation Center of Yangtze River Delta Region Green Pharmaceuticals, College of Pharmaceutical Sciences, Zhejiang University of Technology, Hangzhou 310014, China; zgk7721@163.com (G.Z.); qt866428@163.com (T.G.); 2 College of Biotechnology and Bioengineering, Zhejiang University of Technology, Hangzhou 310014, China; yfyan@zjut.edu.cn; 3 State Key Laboratory of Molecular Engineering of Polymers, Fudan University, 2005 Songhu Road, Shanghai 200433, China

**Keywords:** amphiphilic functional copolymers, click chemistry, self-assembly, biodegradable, low cytotoxicity

## Abstract

Amphiphilic copolymers with pendant functional groups in polyester segments are widely used in nanomedicine. These enriched functionalities are designed to form covalent conjugates with payloads or provide additional stabilization effects for encapsulated drugs. A general method is successfully developed for the efficient preparation of functional biodegradable PEG-polyester copolymers via click chemistry. Firstly, in the presence of mPEG as initiator, Sn(Oct)_2_-catalyzed ring-opening polymerization of the α-alkynyl functionalized lactone with D,L-lactide or ε-caprolactone afforded linear mPEG-polyesters bearing multiple pendant alkynyl groups. Kinetic studies indicated the formation of random copolymers. Through copper-catalyzed azide-alkyne cycloaddition reaction, various small azido molecules with different functionalities to polyester segments are efficiently grafted. The molecular weights, polydispersities and grafting efficiencies of azido molecules of these copolymers were investigated by NMR and GPC. Secondly, it is demonstrated that the resulting amphiphilic functional copolymers with low CMC values could self-assemble to form nanoparticles in aqueous media. In addition, the in vitro degradation study and cytotoxicity assays indicated the excellent biodegradability and low cytotoxicity of these copolymers. This work provides a general approach toward the preparation of functional PEG-polyester copolymers in a quite efficient way, which may further facilitate the application of functional PEG-polyesters as drug delivery materials.

## 1. Introduction

Amphiphilic copolymers with unique properties have been widely used in biomedical applications, including nanomedicine, disease diagnosis, tissue engineering and drug delivery, etc. [1,2,3,4,5,6]. Especially in the development of novel drug delivery systems, amphiphilic copolymers have been extensively investigated. It has been well established that amphiphilic copolymers can self-assemble into core-shell nanostructure with an inner hydrophobic core and a hydrophilic shell that interfaces the aqueous media. Drug delivery systems based on this nanostructure can increase the solubility of highly hydrophobic drugs, improve the stability of protein- and gene-based biotherapeutics, and reduce systemic toxicity of chemotherapeutics [7]. Among these amphiphilic copolymers, PEG-polyester copolymers, such as poly(ethylene glycol)-*b*-polylactide (PEG-PLA), poly(ethylene glycol)-*b-*poly(lactide-co-glycolide) (PEG-PLGA) and poly(ethylene glycol)-*b-*poly(ε-caprolactone) (PEG-PCL), are the most potential materials for drug delivery due to their biodegradability, biocompatibility, tunability and low immunogenicity [8,9,10,11,12]. Normally, these PEG-polyester copolymers are only chemically modifiable at the end of the polymer chains while short of active groups along the skeleton of polyesters. In these traditional PEG-polyester-based drug delivery systems, drugs are usually loaded and stabilized merely via hydrophobic interactions. With the advances in biomedical technology, functional polymer materials with potential biological activities have been widely used in drug delivery systems [13,14,15,16,17]. Recently, it has been demonstrated that introduction of active functional groups onto copolymers successfully afforded novel drug delivery systems with additional stabilization effect between drugs and the carrier polymers, such as π−π interactions [18], donor–acceptor interactions [19], and hydrogen bonding interactions [20]. In addition, multiple reactive groups on the carrier polymers make it possible to prepare polymer–drug conjugates with high drug loading [21]. Therefore, it is highly desirable to prepare versatile PEG-polyester copolymers with pendant functional groups in polyester backbone in a simple and efficient way.

Recent progress in polymerization technology makes it possible to design and synthesize well defined and multi-functional polymers. Developed in the past decade, direct homopolymerization or copolymerization of monomers bearing functional groups is a straightforward strategy to prepare functional polyesters with pendant functional groups, such as amine [22], hydroxyl [23,24] and carboxyl group [25,26,27]. However, this method usually requires multi-step monomer synthesis and further purification procedures [28]. In addition, some functional groups may either completely impede or participate in polymerization, which makes it necessary to introduce additional protective groups before polymerization and remove them after polymerization [29].

Post-polymerization modification is a powerful tool for the synthesis of functional polymers. It is based on the polymerization of monomers with functional groups which are inert under the polymerization conditions but can be directly converted into other active groups in subsequent reactions [30,31,32]. Considering the large number of repeating units in polymer chains and the resulting potentially reduced reactivity of functional groups, highly efficient post-polymerization modification protocol is necessary. “Click chemistry” has been widely used in the modification of polymers due to its high efficiency and selectivity, good tolerance to various chemical groups, and mild reaction conditions. It has been successfully explored for the preparation of functional polymers via post-polymerization modification processes [33,34,35]. Among them, copper-catalyzed azide-alkyne cycloaddition (CuAAC) has been considered as one of the most successful and extensively studied reactions in the construction of well-defined functional polymers [36,37,38,39,40]. It is easy to set up, tolerant to various functional groups, and affords high yield of product with high selectivity. Many examples of grafting functional groups on the polyester backbone based on CuAAC reaction have been reported [41,42,43,44]. In some cases, azido end-capped PEG chains, as hydrophilic moieties, and other functional molecules were simultaneously conjugated to the polyester backbone bearing alkynyl groups, forming amphiphilic comb-type functional copolymers [45,46,47]. However, there are still quite few reports about the construction of functional linear PEG-polyester copolymers via CuAAC reactions based on post-polymerization modification strategy.

As we mentioned above, it is of high interest to construct core-shell nano-drug delivery systems with active functional groups on the hydrophobic moieties of amphiphilic copolymers. This approach can potentially provide these drug delivery systems with extra stabilization effect between carriers and cargos. In order to efficiently build such a drug delivery platform, we hence developed a general method to prepare linear PEG-polyester copolymers bearing various functional pendant groups via ring-opening polymerization (ROP) and click chemistry. A series of well-defined PEG-polyester copolymers bearing multiple alkynyl groups, i.e., monomethoxy poly(ethylene glycol)-*block*-poly(α-propargyl-δ-valerolactone-*co*-lactide) (mPEG-*b*-P(AVL-*co*-LA)) and monomethoxy poly(ethylene glycol)-*block*-poly (α-propargyl-δ-valerolactone-*co*-caprolactone) (mPEG-*b*-P(AVL-*co*-CL)), were first successfully synthesized by ROP in a controlled way. Kinetic studies indicated the formation of random copolymers. Then, through CuAAC reaction various small azido molecules with different functionalities to polyester segments were efficiently grafted. All of these amphiphilic copolymers containing different functional groups could self-assemble into homogeneous nanoparticles, which demonstrated their potential as drug delivery vehicles. In addition, the degradability and cytotoxicity of these copolymers were further evaluated. This study might provide opportunities to further explore potential biomedical applications of PEG-polyester based polymeric materials.

## 2. Results and Discussion

### 2.1. Syntheses of mPEG-b-P(AVL-co-LA) (PPAL) and mPEG-b-P(AVL-co-CL) (PPAC) Copolymers with Pendant Alkynyl Groups

**Scheme 1:**
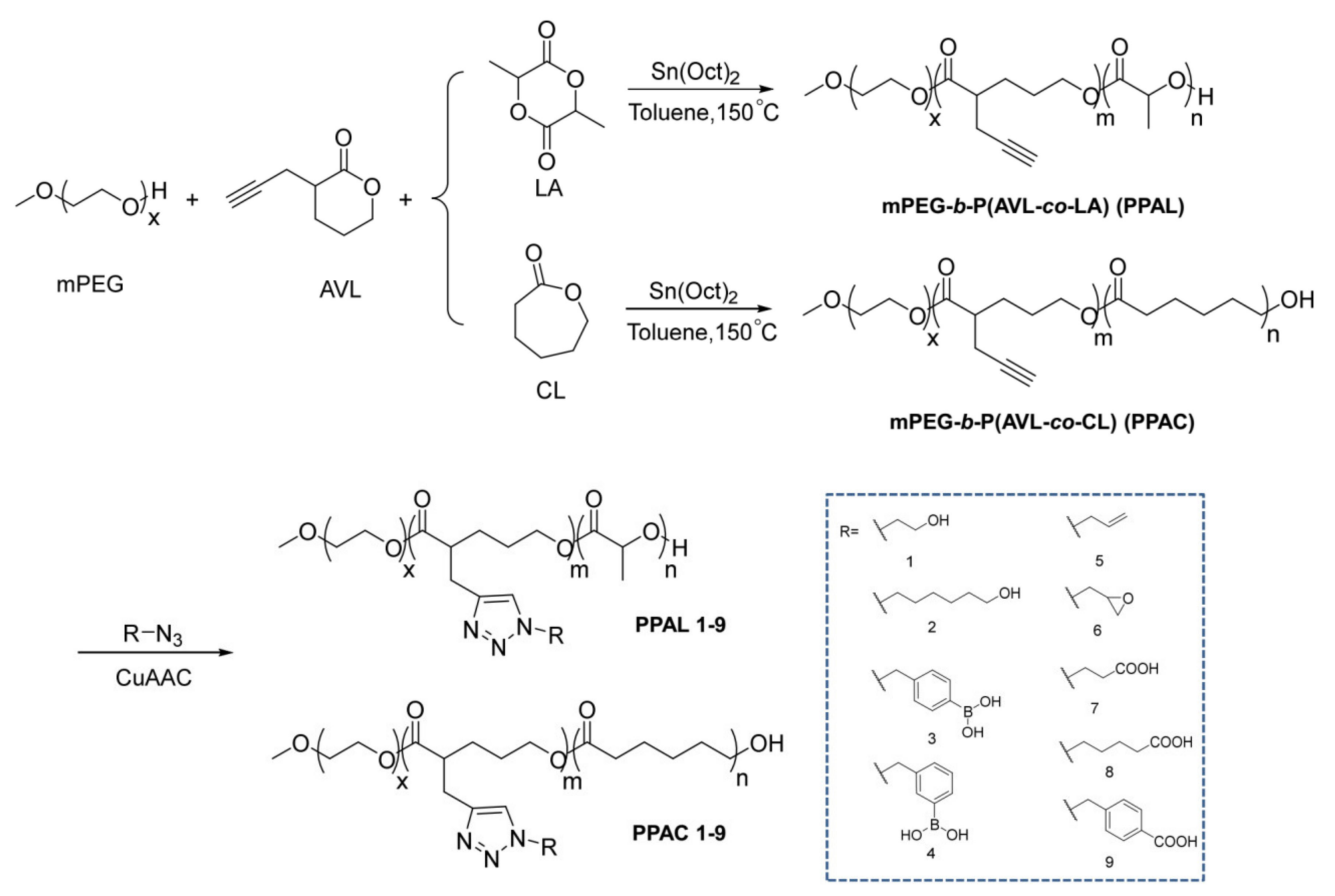
Syntheses and functionalization of **PPAL** and **PPAC** copolymers.

**Figure 1:**
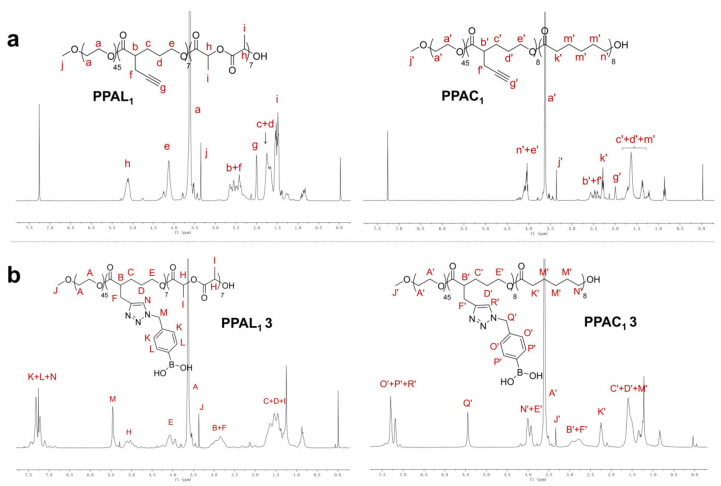
The representative ^1^H NMR of **PPAL_1_** and **PPAC_1_** copolymers (**a**); **PPAL_1_ 3** and **PPAC_1_ 3** copolymers (**b**).

In order to construct multi-functional core-shell nano-drug delivery systems, we developed a modular synthetic approach for the preparation of linear PEG-polyester copolymers bearing various functional pendant groups via ring-opening polymerization (ROP) and click chemistry. First of all, a series of biodegradable amphiphilic **PPAL** and **PPAC** copolymers with different molecular weights and monomer ratios were synthesized. In the presence of mPEG as initiator, Sn(Oct)_2_ catalyzed ring-opening copolymerization of AVL with D,L-LA or ε-caprolactone (CL) monomers successfully afforded **PPAL** and **PPAC** copolymers bearing desired alkynyl groups (Scheme 1). Molecular weight and chemical structure of these copolymers were characterized by gel permeation chromatography (GPC) and ^1^H NMR, respectively (Table 1, Figure S49). As shown in Table 1, the *M*_n, NMR_ of each copolymer was in agreement with *M*_n, GPC_ and the polydispersity (PDI) was relatively low, which indicated that this ROP process could be readily controlled. Representative ^1^H NMR spectra of mPEG_2K_-*b*-P(AVL-*co*-LA)_2K_ (AVL:LA = 1:1) (**PPAL_1_**) and mPEG_2K_-*b*-P(AVL-*co*-CL)_2K_ (AVL:CL = 1:1) (**PPAC_1_**) copolymers are illustrated in Figure 1a. Distinguished peaks at 2.00 ppm clearly showed the presence of alkynyl groups from monomer AVLs. In addition, the characteristic peaks of PLA moieties at 5.12 ppm of **PPAL_1_** and PCL moieties at 2.29 ppm of **PPAC_1_** were also clearly observed. Based on the information of different peak areas in ^1^H NMR spectra, the monomer composition of these block copolymers was calculated, which exactly determined the number of alkynyl groups grafted. The full ^1^H NMR spectra of these copolymers are shown in Figures S2–S9.

**Figure 2:**
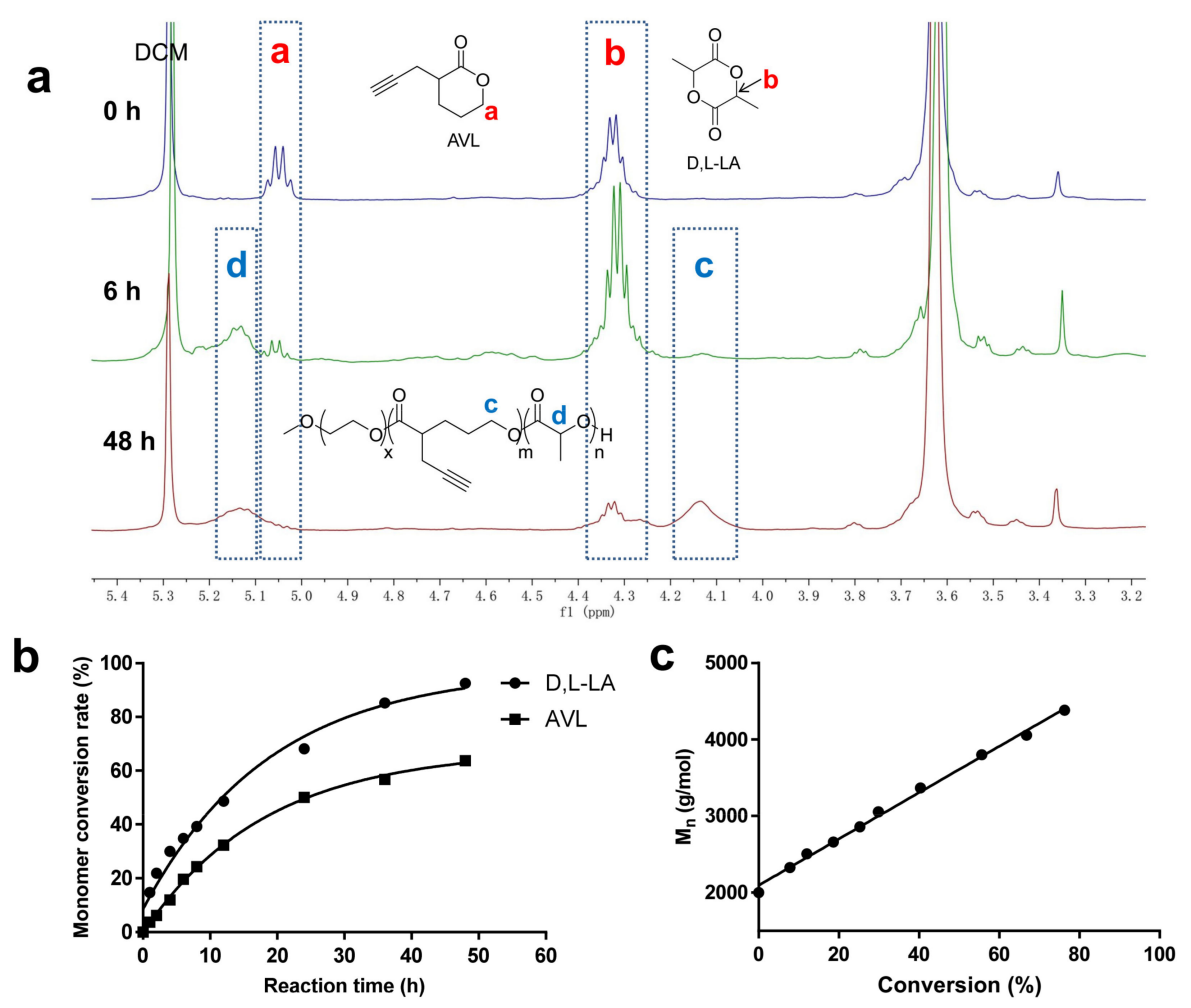
The ^1^H NMR of different reaction time of polymerization of D,L-LA and AVL monomer initiated by mPEG_2K_ (**a**). Plot of monomer conversion in the ring-opening polymerization (**b**). Plot of number-average molecular weight (*M*_n_) of copolymer against total conversion of two monomers (%) in the ring-opening polymerization (**c**). Conditions: [mPEG_2K_] = 0.25 mM, [mPEG_2K_]:[D, L-LA]:[AVL] = 1:7.5:14.5.

As the final applications of copolymer **PPLA** or **PPAC** in drug delivery systems can be significantly affected by its monomer ratio and sequence [48], the living characteristics of the polymerization of D,L-LA and AVL monomers initiated by mPEG_2K_ (conditions: [mPEG_2K_] = 0.25 mM, [mPEG_2K_]:[D,L-LA]:[AVL] = 1:7.5:14.5) were investigated and monitored by ^1^H NMR (in CDCl_3_) and gas chromatography (GC) (in DCM). As revealed in ^1^H NMR spectra (Figure 2a), the characteristic peaks of D,L-LA and AVL monomers gradually disappeared, which indicated that both of the two monomers were consumed simultaneously in polymerization process. Additionally, the progressive formation of resulting copolymers was confirmed (peaks of c and d in Figure 2a). Furthermore, quantitative monitoring of the polymerization of D,L-LA and AVL monomers was determined by GC within 48 h. As shown in Figure 2b, both of two monomers were consumed simultaneously. Compared with AVL, the reactivity of the D,L-LA monomer was higher during the copolymerization process, which might be attributed to the presence of more bulky alkynyl in AVL. As a result, in order to achieve a fairly random copolymer, the feed ratio of AVL in the initial of copolymerization should be higher than in the designed copolymer. In our case, the monomer ratio of D,L-LA to AVL in the formed copolymer chain was basically consistent at a different reaction time and finally a random copolymer chain was obtained when the feed ratio of AVL is 1.9 times that of D,L-LA. Moreover, a linear relationship between the number-average molecular weight (*M*_n_) of copolymer was calculated by ^1^H NMR spectra and the *M*_n_ based on the total conversion of two monomers from GC, which indicated that the copolymerization of D,L-LA and AVL proceeded in a controlled way (Figure 2c).

### 2.2. Functionalization of PPAL and PPAC Copolymers by Click Chemistry

Click chemistry has been widely used to achieve post-polymerization functionalization of polymers due to its high efficiency, excellent selectivity and mild conditions. In this study, the typical copper-catalyzed azide-alkyne cycloaddition (CuAAC) was explored to modify alkyne-containing linear PEG-polyester copolymers with small functional azido molecules, providing a variety of functional PEG-polyester copolymers. The azido molecules (**1–9**) were chosen as functional groups onto the side chains of PPAL or PPAC due to their universality and potential modifiability, which could afford novel drug delivery systems with additional stabilization effect between drugs and carrier polymers [18,19,20,21,49]. Based on these rationales, the functional azido molecules (**1–9**) were synthesized (see Supplementary Materials for details) and then clicked with **PPAL** or **PPAC** copolymers (Scheme 1). To investigate the feasibility of this approach, mPEG_2K_-*b*-P(AVL-*co*-LA)_2K_ (AVL:LA = 1:1) (**PPAL_1_**) and mPEG_2K_-*b*-P(AVL-*co*-CL)_2K_ (AVL:CL = 1:1) (**PPAC_1_**) copolymers were chosen as model polymers to click with azido molecules **1–9**. Under optimized conditions (as illustrated in Scheme 1), all of the nine functional azido molecules were successfully grafted onto copolymers, affording desired functionalized **PPAL_1_ 1–9** and **PPAC_1_ 1–9** (Table 2 and Table S1). The chemical structure of copolymer grafted with functional azido molecules was confirmed by ^1^H NMR. As illustrated in Figure 1b, the representative ^1^H NMR of the resulting **PPAL_1_ 3** and **PPAC_1_ 3** copolymers clearly showed the presence of characteristic peaks of 1, 2, 3-triazole structure and phenyl peak of phenylboronic acid (PBA) structure at 7.48–7.16 ppm, as well as the methylene peak of PBA at 5.46 ppm. Furthermore, the grafting efficiencies (GE %) of the functional group was calculated by integrating the peaks at 5.46 ppm (*I*_M_ or *I*_Q^′^_) (Figure 1b, peak M for PPAL copolymer and peak Q’ for PPAC copolymer) and peaks at 2.00 ppm (*I*_g_ or *I*_g^′^_) (Figure 1a, peak g for PPAL copolymer and peak g’ for PPAC copolymer), respectively (Equations (1) and (2)).

(1) $$GE\;{\left(\%\right)}_{PPAL}=\frac{I_{M}}{2I_{g}}\times 100\%$$

(2) $$GE\;{\left(\%\right)}_{PPAC}=\frac{I_{Q\prime }}{2I_{g\prime }}\times 100\%$$

**Figure 3:**
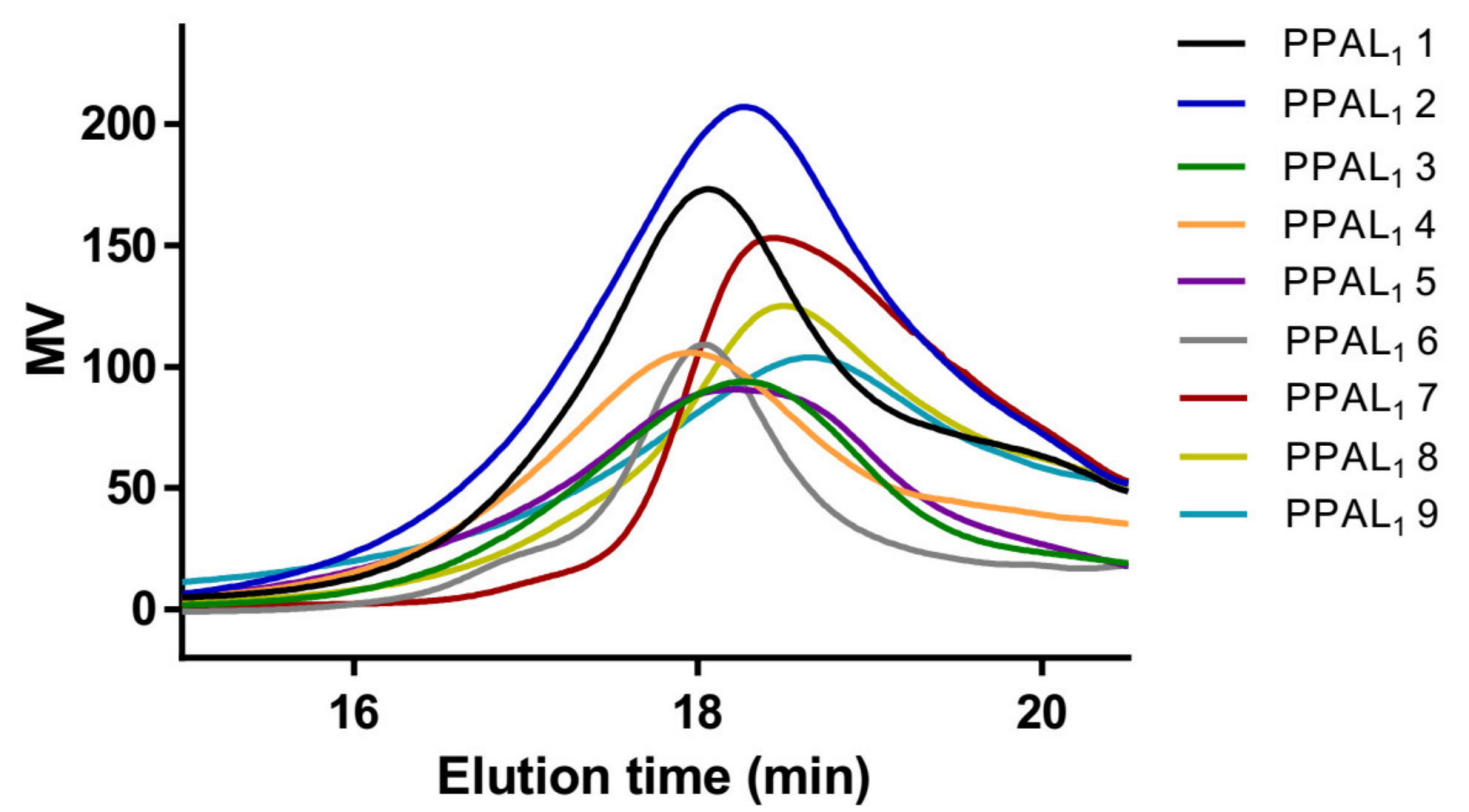
GPC curve of **PPAL_1_ 1–9** copolymers in THF.

For both PPAL and PPAC copolymers, all of these nine functional azido molecules could be efficiently grafted via CuAAC. High grafting efficiencies between 83.6 and 100% were obtained and yields of functionalized copolymers were all above 65.5%. The number- and weight-average molecular weights (*M*_n_ and *M*_w_) and PDI of **PPAL_1_ 1–9** copolymers were determined by ^1^H NMR and GPC (Table 2, Figure 3), the results of which revealed that the functional copolymers with controlled molecular weight and low PDI were synthesized successfully. In addition, CuAAC of azido molecule **3** with **PPAL** and **PPAC** copolymers with different molecular weights and monomer ratios has been investigated, and the results are summarized in Table S2. The grafting efficiencies were between 70.8 and 100% and the yields were all above 76.6%. The full ^1^H NMR spectra of all of these functional copolymers are shown in Figures S31–S48. Moreover, CuAAC of **PPLA_1_ 1** with both azido molecules **3** and **7** was also investigated, and the results revealed that both azido molecules **3** and **7** were successfully grafted on to copolymers (Figure S50). All of these results demonstrated that the post-polymerization functionalization strategy via CuAAC could build a library of versatile polymers in a controlled and efficient way.

### 2.3. Self-Assembly of Functional Polymeric Nanoparticles

**Figure 4:**
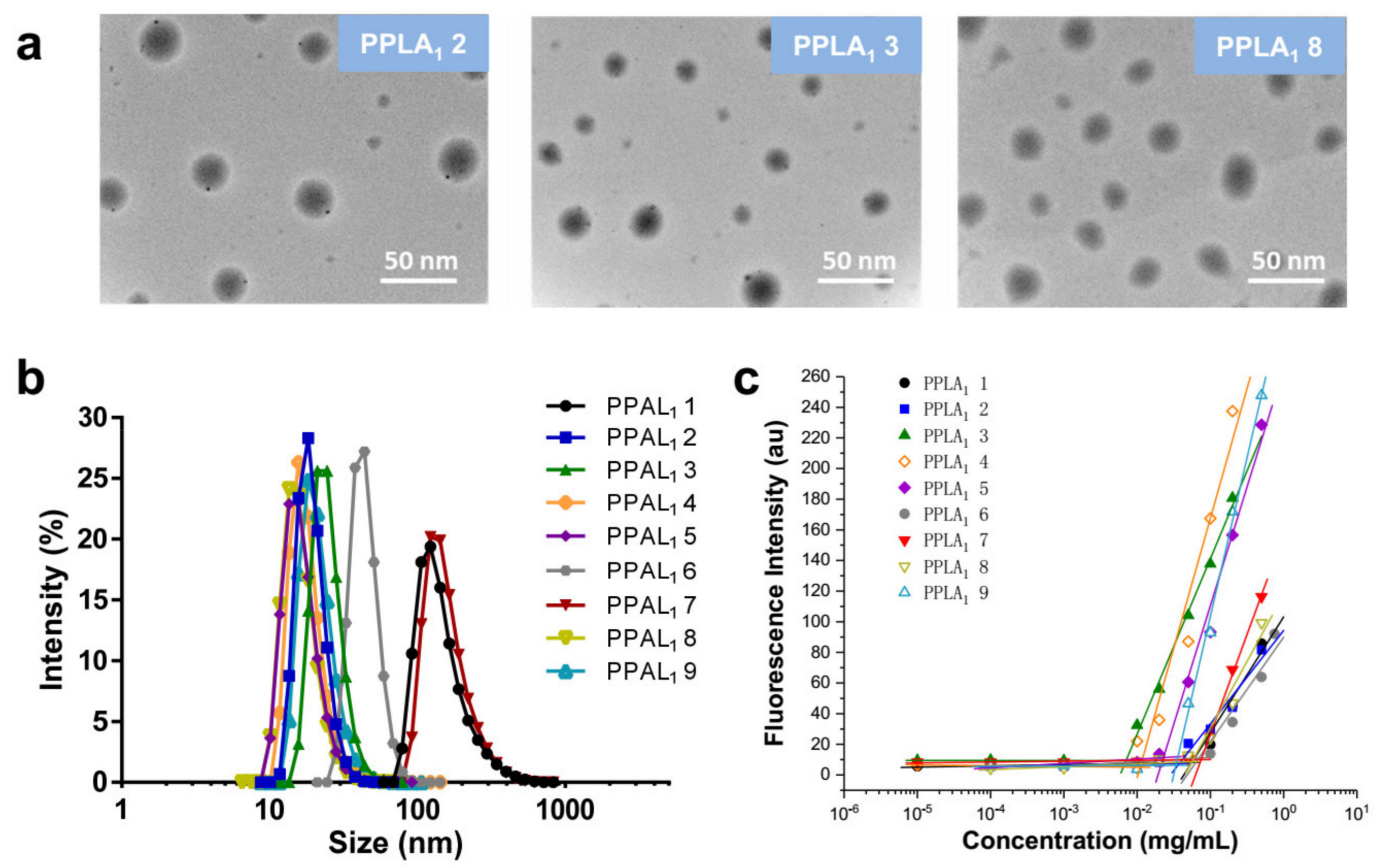
Characterization of related nanoparticles. TEM images of **PPLA_1_ 2**, **PPLA_1_ 3** and **PPLA_1_ 8** nanoparticles (**a**). Dynamic light scattering curves of **PPAL_1_ 1-9** nanoparticles in aqueous media (**b**) and determination of the CMC of **PPAL_1_ 1–9** copolymers (**c**).

It has been well recognized that amphiphilic polymers could self-assemble into nanoparticles (NPs) in aqueous media, which has been extensively exploited for the development of novel nano-drug delivery system [50]. Hence, the ability of functional **PPAL_1_ 1–9** and **PPAC_1_ 1–9** copolymers to form NPs via self-assembly was examined. A nanoprecipitation method was adapted for this test [51]. A solution of functional copolymers in acetone (10 mg mL^−1^) was added dropwise into deionized water, respectively, with vigorous stirring, followed by removal of acetone under vacuum to form NPs. As revealed in TEM images (Figure 4a), uniform spherical structures of the representative **PPLA_1_ 2, PPLA_1_ 3** and **PPLA_1_ 8** NPs were observed. The particle size and polydispersity index (PDI) of **PPAL_1_ 1–9** and **PPAC_1_ 1–9** NPs were measured by dynamic light scattering (DLS). The results are summarized in Table 3 and Table S3 and Figure 4b and Figure S51. Notably, all of these functional copolymers could form NPs with suitable particle sizes for drug delivery applications. Interestingly, the particle sizes of **PPAL_1_ 1**, **PPAL_1_ 7** and **PPAC_1_ 1**, **PPAC_1_ 7** NPs were evidently larger than those of other NPs, which might be attributed to the introduction of hydrophilic hydroxyl groups and carboxyl groups into the polyester segments. It was also found that this influence could be effectively attenuated by extending hydrophobic sections of the azido molecules, which was demonstrated by the smaller particle sizes of NPs based on **PPAL_1_ 2**, **PPAL_1_ 8** and **PPAC_1_** **2**, **PPAC_1_** **8**. This intriguing finding might provide us with an approach to prepare NPs with tunable and even predictable particle size by changing the chemical structure of polymers.

The critical micelle concentration (CMC) is an important parameter of amphiphilic copolymer for drug delivery applications, as it indicates the potential of polymer to form stable micelles under physiological conditions [52]. The CMC of **PPAL_1_ 1–9** copolymers was determined by fluorescence spectroscopy using Nile red as the fluorescent probe. As indicated in Figure 4c and Table 3, the CMC values of **PPAL_1_ 1–9** copolymers were between 0.007 and 0.075 mg mL^−1^. In general, the above results showed the capacity of amphiphilic functional **PPAL_1_ 1–9** copolymers to form stable NPs in aqueous media at low concentration. Interestingly, the CMC value of **PPAL_1_ 1** is higher that of **PPAL_1_ 2** and the same trend was also observed for **PPAL_1_ 7** and **PPAL_1_ 8**. This also might imply a close correlation between CMC and the subtle chemical structure of polymers.

### 2.4. Degradation Study

**Figure 5:**
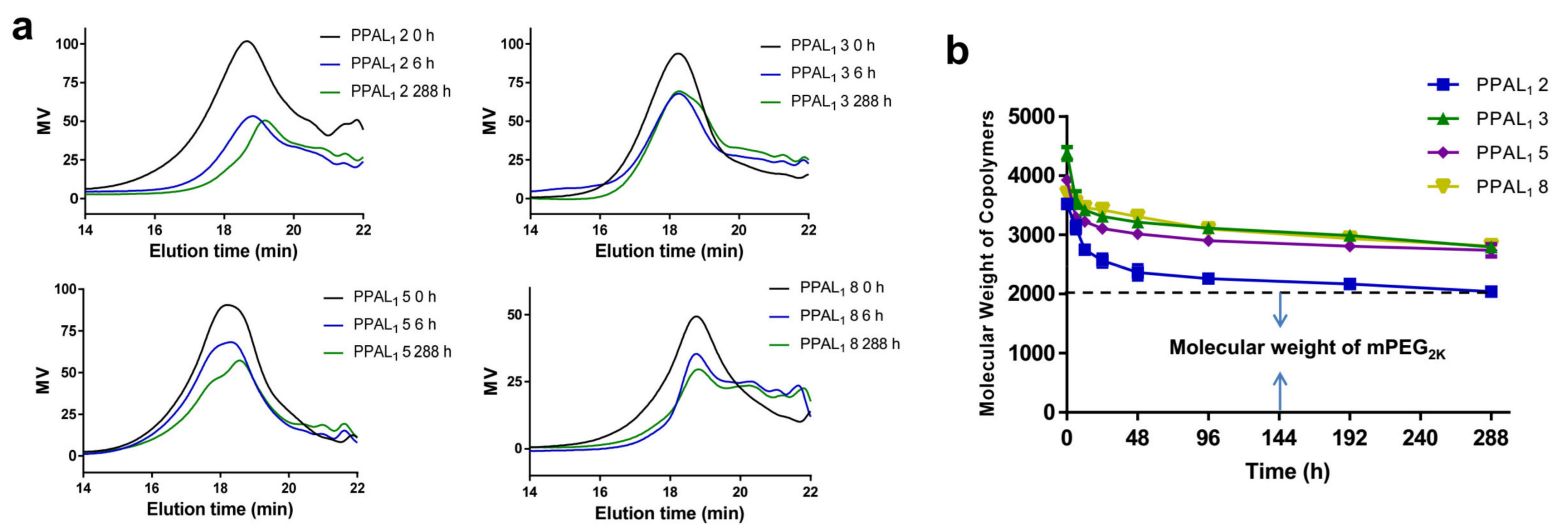
In vitro degradation of the functional copolymers. (**a**) GPC of **PPAL_1_ 2**, **PPAL_1_ 3**, **PPAL_1_ 5** and **PPAL_1_ 8** copolymers at 0 (trace black), 6 (trace blue) and 288 h (trace green) in pH 7.4 PBS at 37 °C. (**b**) The time courses of the in vitro hydrolytic degradation of **PPAL_1_ 2**, **PPAL_1_ 3**, **PPAL_1_ 5** and **PPAL_1_ 8** copolymers during 288 h.

The typical in vitro hydrolytic degradation study of **PPAL_1_ 2**, **PPAL_1_ 3**, **PPAL_1_ 5** and **PPAL_1_ 8** copolymers was performed by monitoring molecular weight change using GPC. As illustrated in Figure 5a, the GPC curves were recorded for the degradation study of **PPAL_1_ 2**, **PPAL_1_ 3**, **PPAL_1_ 5** and **PPAL_1_ 8** copolymers at 0, 6 and 288 h in pH 7.4 PBS at 37 °C. As expected, the formation of low molecular weight degradation products was observed due to the facture of water-susceptible ester bonds in the polyester section of copolymers. In addition, the time courses of the hydrolytic degradation in vitro of copolymers during 288 h are shown in Figure 5b. It was clear that, for all copolymers, a fast degradation rate was observed in the initial 48 h and then the degradation was gradually slowed down in later stages. The molecular weights of polyester section of **PPAL_1_ 2**, **PPAL_1_ 3**, **PPAL_1_ 5** and **PPAL_1_ 8** copolymers were decreased by approximately 53% over the 288 h period. As a result, the biodegradability of these functional copolymers makes them potentially applicable for drug delivery systems.

### 2.5. Cytotoxicity Assay

**Figure 6:**
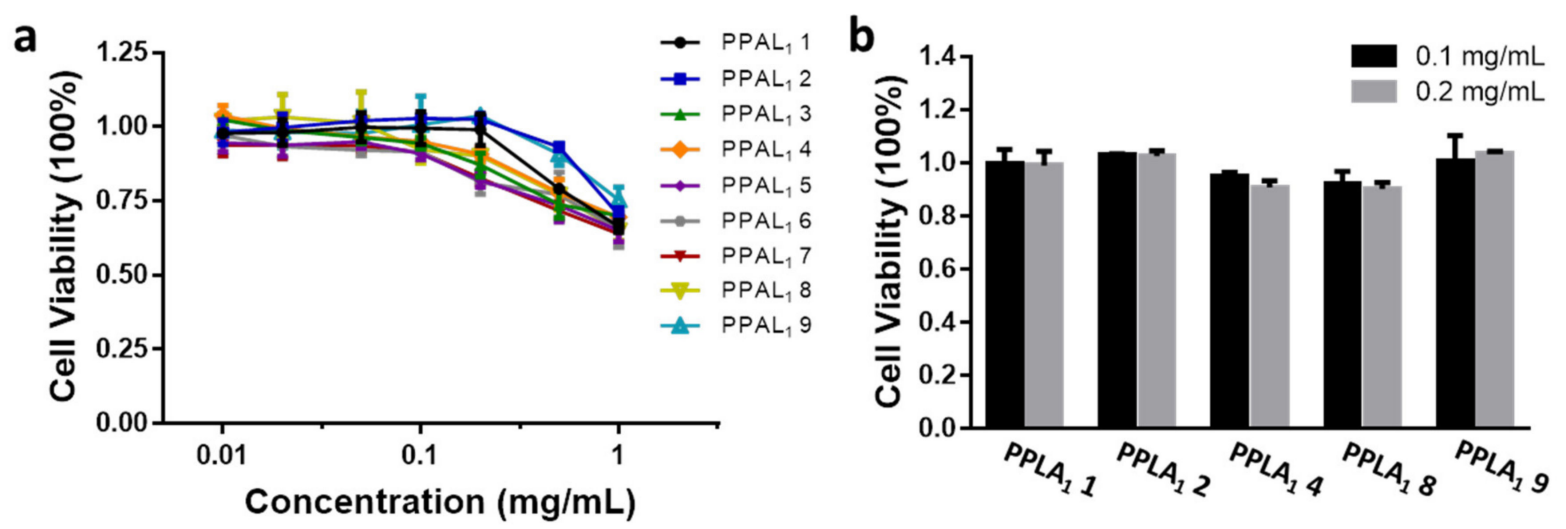
In vitro cytotoxicity of **PPAL_1_ 1–9** copolymers in HeLa cells at various concentrations (**a**) and **PPAL_1_ 1**, **2**, **4**, **8** and **9** copolymers with cell survival up to 90% at 0.2 mg mL^−1^ (**b**).

The in vitro cytotoxicity of **PPAL_1_ 1–9** and **PPAC_1_ 1–9** copolymers in HeLa cells was investigated using MTT method. HeLa cells were treated with copolymers at various concentrations (ranging from 0.01 to 1 mg mL^−1^), and untreated HeLa cells with a cell viability of 100% were used as control. As shown in Figure 6a, after incubation for 24 h, the viability of HeLa cells treated with **PPAL_1_ 1–9** copolymers was over 90%, even if the polymer concentrations were up to 0.1 mg mL^−1^. Moreover, **PPAL_1_ 1**, **2**, **4**, **8** and **9** copolymers showed lower cytotoxicity when the polymer concentration reached 0.2 mg mL^−1^, and the cell survival was up to 90% (Figure 6b). A similar phenomenon was observed in **PPAC_1_ 1–9** copolymers (Figure S52). The results indicated that these functional copolymers were of good biocompatibility with no significant cytotoxicity, making them suitable for biomedical applications.

## 3. Materials and Methods

### 3.1. Materials

Lithium diisopropylamide (2 M sol. In THF/n-heptane/ethylbenzene), N,N,N′,N″,N″-pentamethyldiethylenetriamine (99%), and cuprous bromide (99%) were purchased from Aladdin chemicals (Shanghai, China). mPEGs (*M*_n_ = 2000, 4000) were purchased from TCI chemicals (Shanghai, China). Propargyl bromide (98%), hexamethylphosphoramide (HMPA) (98%), δ-valerolactone (98%), Sn(Oct)_2_ (97%), D,L-lactide (99%), ε-caprolactone (99%), ethylene glycol (99%), 1,6-hexanediol (97%), *p*-toluenesulfonyl chloride (99%), triethylamine (99.5%), DMAP (98%), *p*-tolylboronic acid (98%), *m*-tolylboronic acid (98%), pinacol (99%), *N*-bromosuccinimide (98%), AIBN (98%), KHF_2_ (99%), LiOH·H_2_O (98%), 3-bromopropionic acid (98%), 3-bromoprop-1-ene (98%), epichlorohydrin (99%), *p*-toluic acid (98%), potassium iodide (98%), ethyl 5-bromovalerate (97%), sodium L-ascorbate (98%), and cupric acetate (97%, anhydrous) were purchased from Energy chemicals (Shanghai, China). All solvents were dried before use.

### 3.2. Instruments

^1^H NMR spectra were recorded at 400 MHz (Bruker AVVANCE DRX-400 NMR spectrometer, Bruker, Switzerland). CDCl_3_ and DMSO-*d*_6_ were used as deuterated solvents. Chemical shifts (δ, ppm) were determined with internal solvent signal as reference (CHCl_3_: δ = 7.26, DMSO-*d*_6_: δ = 2.50). Monomer conversion during polymerization was determined based on the concentration of residual monomer using gas chromatography (GC) (FULI9720 series, RBX-5 capillary column 30 m × 0.32 mm × 1.0 μm). The column temperature was 150 °C (15 min). N_2_ was used as the eluent at a flow rate of 1.0 mL min^−1^. Gel permeation chromatography (GPC) (Waters 1515–2414, Waters, Milford, MA, USA) was used to determine the number- and weight-average molecular weights (*M*_n_ and *M*_w_) and polydispersity (*M*_w_/*M*_n_) of copolymers in THF at 30 °C with 1 mL min^−1^ flow rate. Polystyrene standards were used to calibrate the instrument. Transmission electron microscopy (TEM) images were taken with a Hitachi HT7700 instrument operating at an accelerating voltage of 120 KV. The particle size and polydispersity index (PDI) of polymeric nanoparticles were determined by dynamic light scattering (DLS) equipped with a Malvern Nano ZS90 Zetasizer, a 632.8 nm He-Ne laser, and a 173° backscatter detector.

### 3.3. Synthesis of α-Propargyl-δ-valerolactone (AVL)

AVL was synthesized by reported procedures [53]. To a 1 L round-bottom flask charged with 300 mL of anhydrous THF was added lithium diisopropylamide (LDA, 55.1 mL, 110.0 mmol) at −78 °C, followed by dropwise addition of δ-valerolactone solution in anhydrous THF (0.4 M, 250 mL) over 1.5 h with stirring. After addition, the mixture was stirred for another 40 min. Then, a mixture of propargyl bromide (13.4 mL, 120.0 mmol) and hexamethylphosphoramide (20.9 mL, 120.0 mmol) was added dropwise over 40 min and the reaction mixture was warmed up to −30 °C and stirred for another 2 h. The reaction was quenched by adding excess saturated aqueous ammonium chloride solution and then allowed to warm up to room temperature. THF was removed by vacuum and the residue was extracted with ether (3 × 100 mL). The combined organic layers were washed with brine, dried over anhydrous Na_2_SO_4_, filtered, and concentrated under vacuum. Further purification of crude product by flash column chromatography on silica gel (Hexanes:EtOAc = 9:1→6:1) afforded α-propargyl-δ-valerolactone (6.21 g, 45% yield). Higher purity of AVL monomer was obtained by vacuum distillation (5.59 g, 90% yield). ^1^H NMR (400 MHz, CDCl_3_, δ): 4.44–4.23 (m, 2H), 2.78–2.60 (m, 2H), 2.59–2.44 (m, 1H), 2.30 (dd, *J* = 13.5, 7.0 Hz, 1H), 2.03 (t, *J* = 2.6 Hz, 1H), 1.99–1.89 (m, 2H), 1.81–1.66 (m, 1H) (Figure S1).

### 3.4. General Procedure for Synthesis of mPEG-b-P(AVL-co-LA) (PPAL) and mPEG-b-P(AVL-co-CL) (PPAC) Copolymers

All of the glass apparatus for the reaction were flame-dried before use. To a round-bottom flask purged with N_2_ was added a solution of mPEG and monomers in anhydrous toluene. The reaction mixture was stirred at 150 °C for 10 min, followed by the addition of a few drops of Sn(Oct)_2_ catalyst. The polymerization proceeded for another 48 h. After the reaction, the mixture was diluted by 200 mL of dichloromethane (DCM) and acidified by adding a few drops of 0.1 M HCl. It was then washed by H_2_O for several times until the water phase changed to neutral. The organic layer was dried over anhydrous Na_2_SO_4_, filtered, and concentrated under vacuum. The residue was further purified by precipitation from DCM:PE (1:10) for three times and the final product was dried under vacuum at 40 °C overnight. The yields ranged from 63% to 78%. The representative ^1^H NMR of mPEG_2K_-*b*-P(AVL-*co*-LA)_2K_ (AVL:LA = 1:1) (**PPAL_1_**) and mPEG_2K_-*b*-P(AVL-*co*-CL)_2K_ (AVL:CL = 1:1) (**PPAC_1_**) copolymers are shown in Figure 1a.

**PPAL_1_**: ^1^H NMR (400 MHz, CDCl_3_, δ): 5.28–4.99 (m, 14H), 4.36–4.23 (m, 3H), 4.18–4.04 (m, 14H), 3.82–3.41 (m, 182H), 3.35 (s, 3H), 2.69–2.38 (m, 21H), 2.00 (s, 7H), 1.81–1.63 (m, 28H), 1.57–1.41 (m, 42H). GPC (THF, RI): *M*_n_ (PDI) = 3600 g mol^−1^ (1.33).

**PPAC_1_**: ^1^H NMR (400 MHz, CDCl_3_, δ): 4.27–3.98 (m, 34H), 3.82–3.41 (m, 182H), 3.35 (s, 3H), 2.62–2.34 (m, 24H), 2.32–2.26 (m, 16H), 2.00 (s, 8H), 1.76–1.33 (m, 80H). GPC (THF, RI): *M*_n_ (PDI) = 4200 g mol^−1^ (1.47).

The full ^1^H NMR spectra of these copolymers are shown in Figures S2–S9.

### 3.5. General Procedure for Modification of mPEG-b-P(AVL-co-LA) (PPAL) and mPEG-b-P(AVL-co-CL) (PPAC) Copolymers by CuAAC Click Chemistry

**PPAL** or **PPAC** copolymer (1 equiv. in terms of alkyne), azido molecules **1–6** (3 equiv.), cuprous bromide (1 equiv.) and N, N, N′,N″,N″-pentamethyldiethylenetriamine (1 equiv.) were added to anhydrous DMF (4 mL) (for azido molecules **7–9**, the catalytic conditions were modified as 0.1 equiv of cupric acetate and 0.2 equiv. of sodium L-ascorbate). The reaction mixture was stirred in the dark at 50 °C for 24 h under N_2_ atmosphere. After the reaction, H_2_O (4 mL) was added dropwise and then the mixture was dialyzed in H_2_O (molecular weight cutoff of 5 kD) for 48 h. Vacuum freeze-drying afforded pure **PPAL_1_ 1–9** and **PPAC_1_ 1–9** with yields ranging from 65.5% to 93.0%. The representative ^1^H NMR of **PPAL_1_ 3** (**PPAL_1_** modified with azido molecule 3) and **PPAC_1_ 3** (**PPAC_1_** modified with azido molecule 3) copolymers are shown in Figure 1b.

**PPAL_1_ 3**: ^1^H NMR (400 MHz, CDCl_3_, δ): 7.48–7.16 (m, 35H), 5.46 (s, 14H), 5.28–4.85 (m, 14H), 4.34–4.19 (m, 3H), 4.11–3.92 (m, 14H), 3.82–3.41 (m, 182H), 3.37 (s, 3H), 3.13–2.62 (m, 21H), 1.72–1.41 (m, 70H).

**PPAC_1_ 3**: ^1^H NMR (400 MHz, CDCl_3_, δ): 7.48–7.16 (m, 40H), 5.46 (s, 16H), 4.09–3.86 (m, 34H), 3.82–3.41 (m, 182H), 3.33 (s, 3H), 3.00–2.64 (m, 24H), 2.32–2.20 (m, 16H), 2.00 (s, 8H), 1.76–1.33 (m, 80H).

The full ^1^H NMR spectra of these functional copolymers are shown in Figures S31–S48.

### 3.6. Preparation of Polymeric Nanoparticles

Polymeric nanoparticles (NPs) were prepared by a nanoprecipitation method [51]. A solution of copolymer (**PPAL_1_ 1–9** and **PPAC_1_ 1–9**, 20 mg) in acetone (2 mL) was added dropwise into deionized water (10 mL) with stirring. After 20 min, the acetone was removed by a rotary evaporator under reduced pressure, affording aqueous solution of polymeric NPs at a concentration of 2 mg mL^−1^.

### 3.7. Characterization of Polymeric Nanoparticles

The morphological observation of the nanoparticles was performed using a Hitachi HT7700 transmission electron microscopy (TEM). The particle size and polydispersity index (PDI) of **PPAL_1_ 1–9** and **PPAC_1_ 1–9** polymeric NPs were measured by dynamic light scattering (DLS) using a particle size analyzer (Zetasizer, Malvern, UK).

### 3.8. Determination of Critical Micelle Concentration (CMC)

The critical micelle concentration (CMC) of copolymers was characterized by using Nile red as a fluorescence probe [51]. In total, 1 mg of Nile red was dissolved in 30 mL of DCM and then 15 µL of the resulting solution was transferred into several light-proof glass bottles. After evaporation of DCM, 1.5 mL of copolymer aqueous solutions with series concentrations ranging from 0.1 to 75 µg mL^−1^ were added, respectively, into each bottle and stirred at room temperature for 12 h. The fluorescence of Nile red was measured by a microplate reader (Flexstation 3, Molecular Devices LLC, Sunnyvale, CA, USA) with emission wavelength at 620 nm (excitation wavelength at 579 nm).

### 3.9. In Vitro Degradation Study

The in vitro hydrolytic degradation of copolymers was performed under physiological conditions (pH 7.4 PBS buffer, 37 °C) [54]. In total, 25 mg of copolymers was dissolved in 2.5 mL of PBS in Eppendorf tubes, affording a final polymer solution at concentration of 10 mg mL^−1^. The resulting solutions were incubated at 37 °C with stirring and samples of 200 μL were taken from Eppendorf tubes at different time intervals. The samples collected were lyophilized and then dissolved in 200 μL of THF, which were then analyzed by GPC.

### 3.10. Cell Culture

HeLa cells were cultured in DMEM medium supplemented with 10% fetal bovine serum (FBS), streptomycin (100 μg mL^−1^) and penicillin (100 units mL^−1^) in a humid atmosphere with 5% CO_2_ at 37 °C.

### 3.11. Cytotoxicity Assay

The in vitro cytotoxicity of copolymers was measured by MTT (3-(4,5-dimethylthiazol-2-yl)-2,5-diphenyltetrazolium bromide) assays. HeLa cells were seeded in 96-well plates (10,000 cells per well) and incubated at 37 °C. After 12 h, the medium was replaced with 180 µL of fresh medium and then 20 µL of copolymer solutions with series concentrations were added. The incubation continued for another 24 h at 37 °C. At the end of the incubation, 20 µL of MTT solutions (5 mg mL^−1^ in PBS) were added, respectively, to each well. After incubation for 4 h, the medium was removed and 150 µL of DMSO solution was added to dissolve the purple MTT formazan crystals. The cytotoxicity was determined by a microplate reader at 570 nm (Flexstation 3, Molecular Devices LLC, Sunnyvale, CA, USA).

## 4. Conclusions

In summary, a series of novel amphiphilic biodegradable linear PEG-polyester copolymers with various pendant functional groups in polyester segments was successfully synthesized through ROP and click chemistry. The typical CuAAC click reaction proved to be a powerful post-modification tool of the functionalization of polymers with high efficacy and good functional group tolerance. Kinetic studies determined by ^1^H NMR and GC indicated the formation of fairly random copolymers. The molecular weights and PDI values of copolymers determined by ^1^H NMR and GPC showed that these polymers were of controllable properties. In addition, low CMC values were observed for the resulting amphiphilic functional copolymers which could self-assemble to form nanoparticles in aqueous media with controlled particle sizes. Furthermore, the in vitro degradation and cytotoxicity assays of these functional copolymers indicated their good biodegradability and low cytotoxicity, giving them potential for biomedical applications. This work not only provides a promising strategy for the construction of a drug delivery platform based on functional PEG-polyester copolymers but also may extend potential applications of these polymeric materials in biomedical fields.

## Figures and Tables

**Table 1:** Molecular weight and chemical composition of **PPAL** and **PPAC** copolymers via ROP.

Copolymer	[LA] or [CL]/[AVL] Molar Ratio in the Feed	*M*_n,NMR_*^(a)^* (g moL^−1^)	*M*_n,GPC_ *^(b)^* (g moL^−1^)	PDI *^(c)^*	Number of Alkynyl *^(d)^*
mPEG_2K_-*b*-P(AVL-*co*-LA)_2K_ (AVL:LA = 1:1) **(PPAL_1_)**	1/1.9	4100	3600	1.33	7
mPEG_2K_-*b*-P(AVL-*co*-LA)_2K_ (AVL:LA = 1:3) **(PPAL_2_)**	3/1.9	3900	4200	1.31	4
mPEG_4K_-*b*-P(AVL-*co*-LA)_4K_ (AVL:LA = 1:1) **(PPAL_3_)**	1/1.9	7800	6900	1.25	14
mPEG_4K_-*b*-P(AVL-*co*-LA)_4K_ (AVL:LA = 1:3) **(PPAL_4_)**	3/1.9	7600	8000	1.30	7
mPEG_2K_-*b*-P(AVL-*co*-CL)_2K_ (AVL:CL = 1:1) **(PPAC_1_)**	1/1.9	3900	4200	1.27	7
mPEG_2K_-*b*-P(AVL-*co*-CL)_2K_ (AVL:CL = 1:3) **(PPAC_2_)**	3/1.9	3900	4300	1.25	4
mPEG_4K_-*b*-P(AVL-*co*-CL)_4K_ (AVL:CL = 1:1) **(PPAC_3_)**	1/1.9	7500	7000	1.23	12
mPEG_4K_-*b*-P(AVL-*co*-CL)_4K_ (AVL:CL = 1:3) **(PPAC_4_)**	3/1.9	7800	8100	1.31	7

***^(a)^*** Determined from ^1^H NMR spectra; ***^(b,c)^*** Determined by GPC with polystyrene standards; ***^(d)^*** The number of alkynyl groups was calculated based on ^1^H NMR spectra.

**Table 2:** Results of **PPAL_1_** copolymers click with azido molecules **1–9** via CuAAC reaction.

	mPEG_2K_-*b*-P(AVL-*co*-LA)_2K_ (AVL:LA = 1:1) (PPAL_1_)				
Azido Molecule	Grafting Efficiency *^(a)^* (%)	Yield (%)	*M*_n,NMR_ *^(b)^* (g mol^−1^)	*M*_n,GPC_*^(c)^* (g mol^−1^)	PDI *^(d)^*
**1**	98.6	65.5	4100	5800	1.25
**2**	96.7	69.5	5100	5600	1.28
**3**	100.0	82.5	5300	5300	1.28
**4**	93.9	75.0	5100	4400	1.32
**5**	100.0	74.0	5100	5900	1.24
**6**	98.3	85.6	5100	4800	1.14
**7**	86.3	70.0	5100	4900	1.20
**8**	83.6	91.5	4600	5100	1.22
**9**	98.4	70.2	4700	4500	1.22

***^(a)^*** Grafting efficiencies (%) were calculated from the ^1^H NMR spectra; ***^(b)^*** Determined from ^1^H NMR spectra; ***^(c, d)^*** Determined by GPC with polystyrene standards.

**Table 3:** Characterization of **PPAL_1_ 1–9** copolymer nanoparticles.

Functional Copolymer	Particle Size (nm)	PDI	CMC (mg mL^−1^)
PPAL_1_ 1	159.65 ± 5.6	0.153 ± 0.02	0.059
PPAL_1_ 2	18.83 ± 1.2	0.312 ± 0.04	0.037
PPAL_1_ 3	27.27 ± 3.2	0.140 ± 0.02	0.007
PPAL_1_ 4	18.14 ± 3.0	0.259 ± 0.01	0.010
PPAL_1_ 5	16.75 ± 0.7	0.301 ± 0.04	0.023
PPAL_1_ 6	35.13 ± 2.9	0.248 ± 0.05	0.071
PPAL_1_ 7	139.7 ± 5.4	0.141 ± 0.03	0.075
PPAL_1_ 8	16.69 ± 3.3	0.218 ± 0.01	0.058
PPAL_1_ 9	22.09 ± 4.2	0.261 ± 0.02	0.034

## Data Availability

The data presented in this study are available on request from the corresponding author.

## Supplementary Materials

The following are available online at https://www.mdpi.com/article/10.3390/ijms221910407/s1.
