# Supplementary material for: Highly Efficient Modular Construction of Functional Drug Delivery Platform Based on Amphiphilic Biodegradable Polymers via Click Chemistry
Source: Int J Mol Sci. 2021 Sep 27;22(19):10407. doi: 10.3390/ijms221910407 (PMC8508947; doi:10.3390/ijms221910407)
Supplement: Supplementary file 1 [file ijms-22-10407-s001.zip › ijms-1393662-supplementary.pdf]

Supplementary Materials

Highly efficient modular construction of functional drug delivery platform based on amphiphilic bio-degradable polymers via click chemistry

Guangkuo Zhao [1], Tongtong Ge [2], Yunfeng Yan [3, 4], Qi Shuai [2,] and Wei-Ke Su [1,]**

1 Collaborative Innovation Center of Yangtze River Delta Region Green Pharmaceuticals, College of Pharmaceutical Sciences, Zhejiang University of Technology, Hangzhou 310014, China; zgk7721@163.com

2 Collaborative Innovation Center of Yangtze River Delta Region Green Pharmaceuticals, Zhejiang University of Technology, Hangzhou 310014, China; qt866428@163.com

3 College of Biotechnology and Bioengineering, Zhejiang University of Technology, Hangzhou 310014, China; yfyan@zjut.edu.cn

4 State Key Laboratory of Molecular Engineering of Polymers, Fudan University, 2005 Songhu Road, Shanghai 200433, China

* Correspondence: qshuai@zjut.edu.cn (Q.S.); pharmlab@zjut.edu.cn (W.-K.S.)

Table of Contents

1. Experimental Section

2. Supporting Data

Figure S1. ^{1}H NMR spectra of AVL monomer.

Figure S2. ^{1}H NMR spectra of mPEG$_{2K}$-*b*-P(AVL-*co*-LA)$_{2K}$ (AVL:LA =1:1) copolymer.

Figure S3. ^{1}H NMR spectra of mPEG$_{2K}$-*b*-P(AVL-*co*-LA)$_{2K}$ (AVL:LA =1:3) copolymer.

Figure S4. ^{1}H NMR spectra of mPEG$_{2K}$-*b*-P(AVL-*co*-CL)$_{2K}$ (AVL:CL=1:1) copolymer.

Figure S5. ^{1}H NMR spectra of mPEG$_{2K}$-*b*-P(AVL-*co*-CL)$_{2K}$ (AVL:CL=1:3) copolymer.

Figure S6. ^{1}H NMR spectra of mPEG$_{4K}$-*b*-P(AVL-*co*-LA)$_{4K}$ (AVL:LA =1:1) copolymer.

Figure S7. ^{1}H NMR spectra of mPEG$_{4K}$-*b*-P(AVL-*co*-LA)$_{4K}$ (AVL:LA =1:3) copolymer.

Figure S8. ^{1}H NMR spectra of mPEG$_{4K}$-*b*-P(AVL-*co*-CL)$_{4K}$ (AVL:CL=1:1) copolymer.

Figure S9. ^{1}H NMR spectra of mPEG$_{4K}$-*b*-P(AVL-*co*-CL)$_{4K}$ (AVL:CL=1:3) copolymer.

Figure S10. ^{1}H NMR spectra of 2-Hydroxyethyl-*p*-toluenesulfonate (**1a**).

Figure S11. ^{1}H NMR spectra of 2-azidoethanol (**1**).

Figure S12. ^{1}H NMR spectra of 6-hydroxyethyl-*p*-toluenesulfonate (**2a**).

Figure S13. ^{1}H NMR spectra of 6-azidohexan-1-ol (**2**).

Figure S14. ^{1}H NMR spectra of 4,4,5,5-tetramethyl-2-(*p*-tolyl)-1,3,2-

dioxaborolane (**3a**).

Figure S15. ^{1}H NMR spectra of 2-(4-(bromomethyl)phenyl)-4,4,5,5-tetramethyl-1,3,2-dioxaborolane (**3b**).

Figure S16. ^{1}H NMR spectra of 2-(4-(azidomethyl)phenyl)-4,4,5,5-tetramethyl-1,3,2-dioxaborolane (**3c**).

Figure S17. ^{1}H NMR spectra of potassium 4-(azidomethyl)phenyltrifluoroborate (**3d**).

Figure S18. ^{1}H NMR spectra of [4-(azidomethyl)phenyl]boronic acid (**3**).

Figure S19. ^{1}H NMR spectra of 4,4,5,5-Tetramethyl-2-(*m*-tolyl)-1,3,2-dioxaborolane (**4a**).

Figure S20. ^{1}H NMR spectra of 2-(3-(Bromomethyl)phenyl)-4,4,5,5-tetramethyl-1,3,2-dioxaborolane (**4b**).

Figure S21. ^{1}H NMR spectra of 2-(3-(azidomethyl)phenyl)-4,4,5,5-tetramethyl-1,3,2-dioxaborolane (**4c**).

Figure S22. ^{1}H NMR spectra of potassium 3-(azidomethyl)phenyltrifluoroborate (**4d**).

Figure S23. ^{1}H NMR spectra of potassium [3-(azidomethyl)phenyl]boronic acid (**4**).

Figure S24. ^{1}H NMR spectra of 3-azidoprop-1-ene (**5**).

Figure S25. ^{1}H NMR spectra of 2-(azidomethyl)oxirane (**6**).

Figure S26. ^{1}H NMR spectra of 3-azidopropanoic acid (**7**).

Figure S27. ^{1}H NMR spectra of ethyl 5-azidopentanoate (**8a**).

Figure S28. ^{1}H NMR spectra of 5-azidopentanoic acid (**8**).

Figure S29. ^{1}H NMR spectra of 4-(bromomethyl)benzoic acid (**9a**).

Figure S30. ^{1}H NMR spectra of 4-(azidomethyl)benzoic acid (**9**).

Figure S31. ^{1}H NMR spectra of **PPAL$_1$ 1** copolymer.

Figure S32. ^{1}H NMR spectra of **PPAC$_1$ 1** copolymer.

Figure S33. ^{1}H NMR spectra of **PPAL$_1$ 2** copolymer.

Figure S34. ^{1}H NMR spectra of **PPAC$_1$ 2** copolymer.

Figure S35. ^{1}H NMR spectra of **PPAL$_1$ 3** copolymer.

Figure S36. ^{1}H NMR spectra of **PPAC$_1$ 3** copolymer.

Figure S37. ^{1}H NMR spectra of **PPAL$_1$ 4** copolymer.

Figure S38. ^{1}H NMR spectra of **PPAC$_1$ 4** copolymer.

Figure S39. ^{1}H NMR spectra of **PPAL$_1$ 5** copolymer.

Figure S40. ^{1}H NMR spectra of **PPAC$_1$ 5** copolymer.

Figure S41. ^{1}H NMR spectra of **PPAL$_1$ 6** copolymer.

Figure S42. ^{1}H NMR spectra of **PPAC$_1$ 6** copolymer.

Figure S43. ^{1}H NMR spectra of **PPAL$_1$ 7** copolymer.

Figure S44. ^{1}H NMR spectra of **PPAC$_1$ 7** copolymer.

Figure S45. ^{1}H NMR spectra of **PPAL$_1$ 8** copolymer.

Figure S46. ^{1}H NMR spectra of **PPAC$_1$ 8** copolymer.

Figure S47. ^{1}H NMR spectra of **PPAL$_1$ 9** copolymer.

Figure S48. ^{1}H NMR spectra of **PPAC$_1$ 9** copolymer.

Figure S49. GPC curve of **PPAL** and **PPAC** copolymers in THF at 30°C with 1 mL/min flow rate.

Figure S50. ^{1}H NMR spectra of **PPAL$_1$ 3, 7** copolymer.

Figure S51. Dynamic light scattering curves of **PPAC$_1$ 1-9** nanoparticles in aqueous media.

Figure S52. *In vitro* cytotoxicity of **PPAC$_1$ 1-9** copolymers in HeLa cells at various concentrations.

Table S1. Results of **PPAC$_1$** copolymers click with azido molecules via CuAAC reaction.

Table S2. Functionalization of **PPAL** and **PPAC** copolymers with azido molecules **3** via CuAAC reaction.

Table S3. Characterization of **PPAC$_1$ 1-9** copolymer nanoparticles.

3. References

1. Experimental Section

Synthesis of Azido Molecules 1-9.

Synthesis of 2-azidoethanol (azido molecules **1).**[S1]

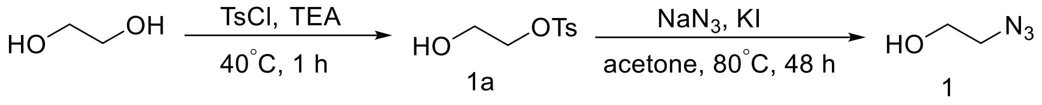

2-Hydroxyethyl-p-toluenesulfonate **(1a):** *p*-Toluenesulfonyl chloride (5.0 g, 26.2 mmol) and ethylene glycol (32.5 g, 524.0 mmol) were mixed in a 250 mL round-bottom flask and stirred at room temperature for 30 min, followed by slow addition of triethylamine (3.65 mL, 26.2 mmol) over 3 min. The mixture was stirred at 40 °C for another 1 h. After reaction, the mixture was diluted by 300 mL of DCM and washed with 300 mL of H_2O. The water phase was then extracted by another 300 mL of DCM. The combined organic layers were dried over anhydrous Na_2SO_4, filtered, and concentrated under vacuum. Further purification of crude product by flash column chromatography on silica gel (Hexanes:EtOAc = 1:1) afforded 2-hydroxyethyl-*p*-toluenesulfonate (4.82 g, 85% yield). **^{1}H NMR** (400 MHz, $CDCl_3$, δ): 7.79 (d, *J* = 8.4 Hz, 2H), 7.34 (d, *J* = 8.4 Hz, 2H), 4.13 (t, *J* = 4.4 Hz, 2H), 3.80 (t, *J* = 4.4 Hz, 2H), 2.44 (s, 3H), 2.21 (s, 1H).

2-Azidoethanol **(1):** To a round-bottom flask charged with a solution of 2-hydroxyethyl-*p*-toluenesulfonate (4.82 g, 22.3 mmol) and KI (0.37 g, 2.2 mmol) in acetone (50 mL) was added a solution of NaN_3 (1.74 g, 26.8 mmol) in H_2O (10 mL). The reaction mixture was stirred at 80 °C for 48 h. After reaction, the organic solvent was evaporated under vacuum and the resulting mixture was extracted with DCM (3×100 ml). The combined organic layers were dried over Na_2SO_4, filtered, and concentrated under vacuum to afford 2-azidoethanol without further purification (1.21 g, 66.3% yield). **^{1}H NMR** (400 MHz, $CDCl_3$, δ)： 3.76 (t, *J* = 4.8 Hz, 2H), 3.42 (t, *J* = 4.4 Hz, 2H), 2.35 (s, 3H).

Synthesis of 6-azidohexan-1-ol (azido molecules **2).**[S2]

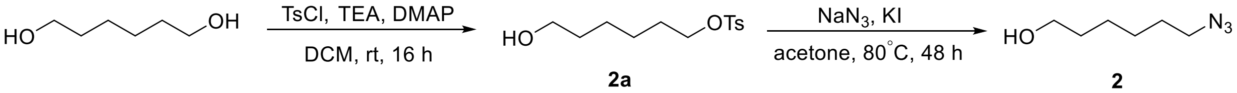

6-Hydroxyethyl-p-toluenesulfonate **(2a):** To a solution of 1,6-hexanediol (5.0 g, 42.3 mmol) and TEA (1.29 mL, 9.3 mmol) in DCM (100 mL) was added *p*-toluenesulfonyl chloride (1.75 g, 8.46 mmol) and DMAP (51 mg, 0.42 mmol). The reaction mixture was stirred at room temperature for 16 h. After reaction, the mixture was diluted by 100 mL of DCM and washed with H_2O (3×50 mL). The organic layer was dried over Na_2SO_4, filtered, and concentrated under vacuum. Further purification of crude product by flash column chromatography on silica gel (Hexanes:EtOAc = 2:1 (v/v)) afforded 6-hydroxyethyl-*p*-toluenesulfonate (1.88 g, 75% yield). **^{1}H NMR** (400 MHz, $CDCl_3$, δ)： 7.77 (d, *J* = 8.4 Hz, 2H), 7.33 (d, *J* = 8.4 Hz, 2H), 4.01 (t, *J* = 6.4 Hz, 2H), 3.59 (t, *J* = 6.4 Hz, 2H), 2.44 (s, 3H), 1.64 (quint, *J* = 6.8 Hz, 2 H), 1.60 (s, 1H,-OH), 1.50 (quint, *J* = 6.8 Hz, 2 H), 1.36-1.27 (m, 4H).

6-Azidohexan-1-ol **(2):** To a round-bottom flask charged with a solution of 6-hydroxyethyl-*p*-toluenesulfonate (1.88 g, 6.9 mmol) and KI (0.12 g, 0.7 mmol) in acetone (50 mL) was added a solution of NaN_3 (0.67 g, 10.4 mmol) in H_2O (10 mL). The reaction mixture was stirred at 80 °C for 48 h. After reaction, the organic solvent was evaporated under vacuum and the resulting mixture was extracted with DCM (3×100 ml). The combined organic layers were dried over Na_2SO_4, filtered, and concentrated under vacuum to afford 6-azidohexan-1-ol without further isolation (0.81 g, 82% yield). **^{1}H NMR** (400 MHz, $CDCl_3$, δ)： 3.59 (t, *J* = 6.4 Hz, 2H), 3.24 (t, *J* = 7.2 Hz, 2H), 2.09 (s, 1H, -OH), 1.64-1.46 (m, 4H), 1.43-1.29 (m, 4H).

Synthesis of [4-(azidomethyl)phenyl]boronic acid (azido molecules **3).**[S3, S4]

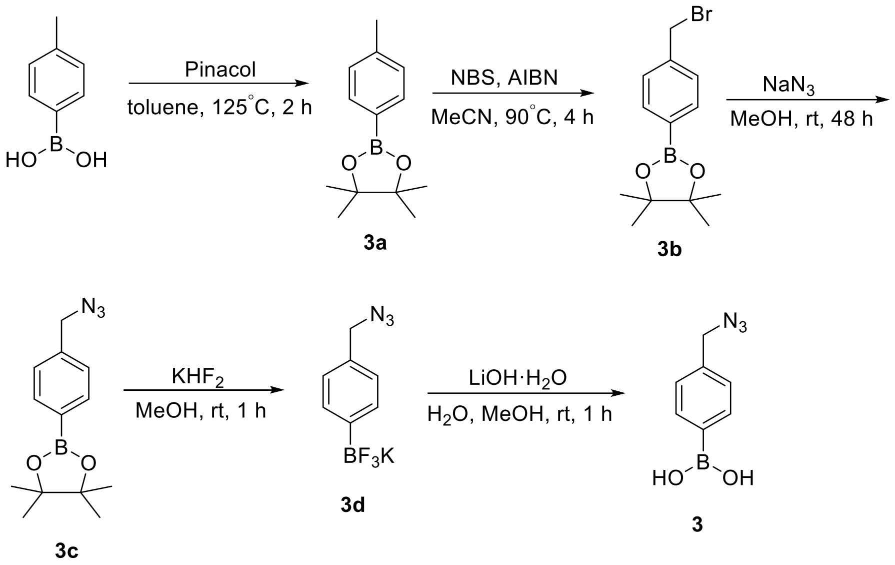

4,4,5,5-Tetramethyl-2-(p-tolyl)-1,3,2-dioxaborolane **(3a)***:* A solution of *p*-tolylboronic acid (5.0 g, 36.8 mmol) and pinacol (5.22 g, 44.2 mmol) in anhydrous toluene (100 mL) was refluxed at 140 °C to remove water using Dean-Stark apparatus and then stirred at 125 °C for another 2 h. After reaction, the organic solvent was evaporated under vacuum. The residue was dissolved in ethyl acetate (100 mL) and washed with H_2O (2×50 mL). The organic layer was dried over Na_2SO_4, filtered, and concentrated under vacuum to afford 4,4,5,5-tetramethyl-2-(*p*-tolyl)-1,3,2-dioxaborolane (7.53 g, 94% yield). **^{1}H NMR** (400 MHz, $CDCl_3$, δ)： 7.72 (d, *J* = 7.6 Hz, 2H), 7.20 (d, *J* = 7.6 Hz, 2H), 2.38 (s, 3H), 1.35 (s, 12H).

2-(4-(Bromomethyl)phenyl)-4,4,5,5-tetramethyl-1,3,2-dioxaborolane **(3b):** A solution of 4,4,5,5-tetramethyl-2-(*p*-tolyl)-1,3,2-dioxaborolane (7.53 g, 34.5 mmol), *N*-bromosuccinimide (4.48 g, 38.0 mmol), and AIBN (113.3 mg, 2 mol%) in acetonitrile (100 mL) was stirred at 90 °C for 4 h. After reaction, acetonitrile was removed under vacuum. The residue was dissolved in hexane (30 mL), followed by filtration and concentration. Recrystallization of crude product from hexane afforded 2-(4-(bromomethyl)phenyl)-4,4,5,5-tetramethyl-1,3,2-dioxaborolane (7.59 g, 74% yield). **^{1}H NMR** (400 MHz, $CDCl_3$, δ)： 7.79 (d, *J* = 8.0 Hz, 2H), 7.40 (d, *J* = 8.0 Hz, 2H), 4.40 (s, 2H), 1.34 (s, 12H).

2-(4-(Azidomethyl)phenyl)-4,4,5,5-tetramethyl-1,3,2-dioxaborolane **(3c):** A solution of 2-(4-(bromomethyl)phenyl)-4,4,5,5-tetramethyl-1,3,2-dioxaborolane (7.59 g, 25.6 mmol) and NaN_3 (2.50 g, 38.4 mmol) in ethanol (100 mL) was stirred at room temperature for 48 h. After reaction, the organic sol-

vent was evaporated under vacuum. The resulting residue was diluted by hexane (100 mL) and washed with H_2O (2×10 ml). The organic layer was dried over Na_2SO_4, filtered, and concentrated under vacuum to afford 2-(4-(azidomethyl)phenyl)-4,4,5,5-tetramethyl-1,3,2-dioxaborolane (5.69 g, 86% yield). **^{1}H NMR** (400 MHz, $CDCl_3$, δ)： 7.84 (d, *J* = 7.6 Hz, 2H), 7.32 (d, *J* = 7.6 Hz, 2H), 4.34 (s, 2H), 1.35 (s, 12H).

Potassium 4-(azidomethyl)phenyltrifluoroborate **(3d):** A suspension of 2-(4-(Azidomethyl)phenyl)-4,4,5,5-tetramethyl-1,3,2-dioxaborolane (5.69 g, 22.0 mmol) and KHF_2 (7.79 g, 132.0 mmol) in methanol (50 mL) was stirred at room temperature for 1 h. After reaction, the organic solvent was evaporated under vacuum. The residue was dissolved in hot acetone (50 mL), followed by filtration and concentration. Recrystallization of crude product from hot acetone and cold ethyl ether afforded potassium 4-(azidomethyl)phenyltrifluoroborate (4.52 g, 86% yield). **^{1}H NMR** (400 MHz, DMSO-d_6, δ)： 7.35 (d, *J* = 9.6 Hz, 2H), 7.09 (d, *J* = 9.6 Hz, 2H), 4.30 (s, 2H).

[4-(Azidomethyl)phenyl]boronic acid **(3):** A solution of potassium 4-(azidomethyl)phenyltrifluoroborate (2.26 g, 9.5 mmol) and $LiOH·H_2O$ (2.78 g, 66.2 mmol) in H_2O (50 mL) and acetonitrile (100 mL) was stirred at room temperature for 24 h. After reaction, saturated aqueous ammonium chloride (80 mL) and hydrochloric acid (1M, 20 mL) were added into the mixture and stirring continued for another 5 min. The mixture was then extracted with ethyl acetate (3×100 mL), and the combined organic layers were dried over Na_2SO_4, filtered, and concentrated under vacuum to afford [4-(azidomethyl)phenyl]boronic acid (1.52 g, 91% yield). **^{1}H NMR** (400 MHz, DMSO-d_6, δ)： 7.82 (d, *J* = 7.6 Hz, 2H), 7.33 (d, *J* = 7.6 Hz, 2H), 4.44 (s, 2H).

Synthesis of [3-(azidomethyl)phenyl]boronic acid (azido molecules **4).**[S3, S4]

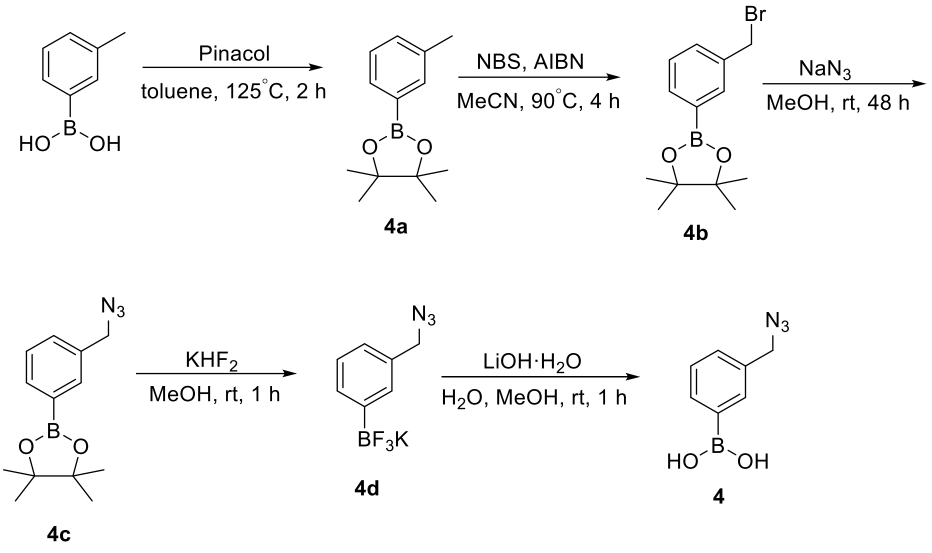

4,4,5,5-Tetramethyl-2-(m-tolyl)-1,3,2-dioxaborolane **(4a):** A solution of *m*-tolylboronic acid (5.0 g, 36.8 mmol) and pinacol (5.22 g, 44.2 mmol) in anhydrous toluene (100 mL) was refluxed at 140°C to remove water using Dean-Stark apparatus and then stirred at 125 °C for another 2 h. After reaction, the organic solvent was evaporated under vacuum. The residue was dissolved in ethyl acetate (100 mL) and washed with H_2O (2×50 ml). The organic layer was dried over Na_2SO_4, filtered, and concentrated under vacuum to afford 4,4,5,5-tetramethyl-2-(*m*-tolyl)-1,3,2-dioxaborolane (6.02 g, 75% yield). **^{1}H NMR** (400 MHz, $CDCl_3$, δ) : 7.76-7.63 (m, 2H), 7.32 (d, *J* = 5.6 Hz, 2H), 2.42 (s, 3H), 1.41 (s, 12H).

2-(3-(Bromomethyl)phenyl)-4,4,5,5-tetramethyl-1,3,2-dioxaborolane **(4b):** A solution of 4,4,5,5-tetramethyl-2-(*m*-tolyl)-1,3,2-dioxaborolane (6.02 g, 20.3 mmol), *N*-bromosuccinimide (2.64 g, 22.4 mmol), and AIBN (66.67 mg, 2 mol%) in acetonitrile (100 mL) was stirred at 90 °C for 4 h. After reaction, the organic solvent was evaporated under vacuum. The residue was dissolved in hexane (30 mL), followed by filtration and concentration. Recrystallization of crude product from hexane afforded 2-3-(bromomethyl)phenyl)-4,4,5,5-tetramethyl-1,3,2-dioxaborolane (6.15 g, 75% yield). **^{1}H NMR** (400 MHz, $CDCl_3$, δ) : 7.87-7.71 (m, 2H), 7.54-7.34 (m, 2H), 4.51 (s, 2H), 1.35 (s, 12H).

2-(3-(Azidomethyl)phenyl)-4,4,5,5-tetramethyl-1,3,2-dioxaborolane **(4c):** 2-(3-(Bromomethyl)phenyl)-4,4,5,5-tetramethyl-1,3,2-dioxaborolane (6.15g, 20.7 mmol) and NaN_3 (2.02 g, 31.1 mmol) were dissolved in 100 mL of ethanol. The reaction mixture was stirred at room temperature for 48 h. After reaction, the organic solution was evaporated under vacuum. The resulting residue was diluted

by 100 mL of hexane and washed with H_2O (2×10 ml). The organic layer was dried over Na_2SO_4, filtered, and concentrated under vacuum to afford 2-(3-(azidomethyl)phenyl)-4,4,5,5-tetramethyl-1,3,2-dioxaborolane (5.20 g, 97% yield). **^{1}H NMR** (400 MHz, $CDCl_3$, δ) : 7.84-7.71 (m, 2H), 7.47-7.36 (m, 2H), 4.35 (s, 2H), 1.35 (s, 12H).

Potassium 3-(azidomethyl)phenyltrifluoroborate **(4d):** 2-(3-(Azidomethyl)phenyl)-4,4,5,5-tetramethyl-1,3,2-dioxaborolane (5.20 g, 20.0 mmol) and KHF_2 (7.08 g, 120.0 mmol) were mixed in 50 mL of methanol. The resulting white suspension was stirred at room temperature for 6 h. After reaction, the organic solution was evaporated under vacuum. The residue was mixed with 50 mL of hot acetone and the remaining solid impurities were removed by filtration. The filtrate was then concentrated under vacuum and further purified by recrystallised from minimal hot acetone and cold ethyl ether to afford potassium 3-(azidomethyl)phenyltrifluoroborate (2.73 g, 57% yield). **^{1}H NMR** (400 MHz, DMSO-d_6, δ) : 7.37-7.26 (m, 2H), 7.17-7.00 (m, 2H), 4.31 (s, 2H).

[3-(Azidomethyl)phenyl]boronic acid **(4):** Potassium 3-(azidomethyl)phenyltrifluoroborate (2.73 g, 11.47 mmol) and LiOH·H_2O (3.36 g, 80.0 mmol) were dissolved in H_2O (50 mL) and acetonitrile (100 mL). The reaction mixture was stirred at room temperature for 24 h. After reaction, saturated aqueous ammonium chloride (80 mL) and 1 M hydrochloric acid (20 mL) were added into reaction mixture and stirred for another 5 min. The mixture was then extracted with ethyl acetate (3×100 mL), and the combined organic layers were dried over Na_2SO_4, filtered, and concentrated under vacuum to afford [4-(azidomethyl)phenyl]boronic acid (1.92 g, 95% yield). **^{1}H NMR** (400 MHz, DMSO-d_6, δ) : 7.86-7.72 (m, 2H), 7.42-7.33 (m, 2H), 4.43 (s, 2H).

Synthesis of 3-azidoprop-1-ene (azido molecules **5).**[S5]

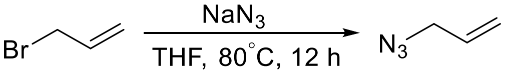

5

3-azidoprop-1-ene **(5):** To a solution of 3-bromoprop-1-ene (1.0 g, 8.3 mmol) in THF (0.5 mL) in a Schlenk tube was added a solution of NaN_3 (8.09 g, 12.5 mmol) in H_2O (2.5 mL) and the reaction mixture was stirred at 80 °C for 12 h. After reaction was completed, the resulting upper layer of mixture was sepa-

rated by using separating funnel and washed with H_2O (2×2 ml). The crude product was dried over Na_2SO_4 and filtered to afford 3-azidoprop-1-ene (0.50 g, 72% yield). **^{1}H NMR** (400 MHz, $CDCl_3$, δ): 5.95-5.80 (m, 1H), 5.38-5.22 (m, 2H), 3.77 (d, J = 6.0 Hz, 2H).

Synthesis of 2-(azidomethyl)oxirane (azido molecules **6).**

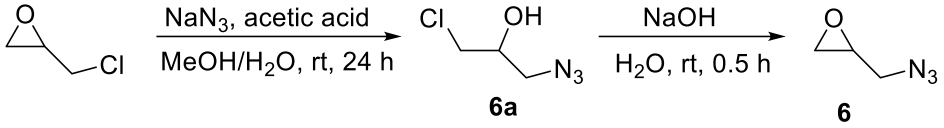

2-(Azidomethyl)oxirane was synthesized by reported procedures.[S6]

1-Azido-3-chloropropan-2-ol **(6a):** A solution of epichlorohydrin (8.0 mL, 102 mmol), sodium azide (8 g, 122 mmol) and acetic acid (7 mL, 122 mmol) in cosolvent of ethanol/water (1:4, 40 mL) was stirred at room temperature for 24 h. After reaction, brine (50 mL) was added, and the mixture was extracted with EtOAc (3x 100 mL). The combined organic layers were dried over Na_2SO_4, filtered, and concentrated under vacuum to afford to 1-azido-3-chloropropan-2-ol (13.19 g, 96% yield). **^{1}H NMR** (400 MHz, $CDCl_3$, δ): 3.99 (pent, J = 5.5 Hz, 1 H), 3.66-3.56 (m, 2 H), 3.48 (d, J = 5.3 Hz, 2 H).

2-(Azidomethyl)oxirane **(6):** 1-Azido-3-chloropropan-2-ol (13.19 g, 97.3 mmol) was added to a solution of NaOH (1 N, 100 mL) and the resulting mixture was stirred at room temperature for 30 min. After reaction, the mixture was extracted with DCM (3×50 ml). The combined organic layers were washed with brine, dried over Na_2SO_4, filtered, and concentrated under vacuum to afford to 2-(azidomethyl)oxirane (8.31 g, 86.2 yield). **^{1}H NMR** (400 MHz, $CDCl_3$, δ): 3.54 (dd, J = 13.8, 3.2 Hz, 1 H), 3.29 (dd, J = 13.5, 5.4 Hz, 1 H), 3.18 (m, 1 H), 2.82 (dd, J = 4.7, 4.1 Hz, 1 H), 2.69 (dd, J = 4.8, 2.5 Hz, 1 H).

Synthesis of 3-azidopropanoic acid (azido molecules **7).**[S7]

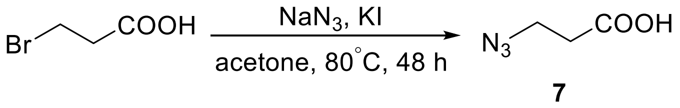

3-Azidopropanoic acid **(7):** 3-Bromopropionic acid (3.0 g, 19.6 mmol) and KI (0.34 g, 2.0 mmol) were dissolved in acetone (50 mL) and the solution of NaN_3 (1.91 g, 29.4 mmol) in H_2O (10 mL) was added. The reaction mixture was stirred at 80 °C for 48 h. After reaction, the organic solution was evaporated un-

der vacuum and the resulting mixture extracted into DCM (3×100 ml). The combined organic layers were dried over Na_2SO_4, filtered, and concentrated under vacuum to afford 3-azidopropanoic acid (1.65 g, 73% yield). **^{1}H NMR** (400 MHz, $CDCl_3$, δ): 3.59 (t, *J* = 8 Hz, 2H), 2.64 (t, *J* = 4.4 Hz, 2H).

Synthesis of 5-azidopentanoic acid (azido molecules **8).**[S8]

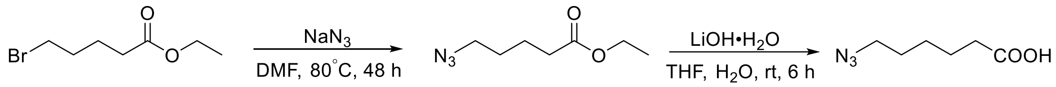

8a
8

Ethyl 5-azidopentanoate **(8a):** Ethyl 5-bromovalerate (5.0 g, 23.9 mmol) and NaN_3 (7.0 g, 107.6 mmol) were added in 50 mL of anhydrous DMF. The reaction mixture was stirred at 80 °C for 48 h. After reaction, the mixture was diluted by hexane/EtOAc (1:4, 200 mL) and washed with saturated $NaHCO_3$ (2 x 150 mL) and H_2O (100 mL). The organic layer was dried over Na_2SO_4, filtered, and concentrated under vacuum to afford ethyl 5-azidopentanoate (3.64 g, 89% yield). **^{1}H NMR** (400 MHz, $CDCl_3$, δ): 4.12 (q, *J* = 7.2 Hz, 2H), 3.28 (t, *J* = 6.4 Hz, 2H), 2.32 (t, *J* = 7.2 Hz, 2H), 1.75-1.57 (m, 4H), 1.25 (t, *J* = 6.8 Hz, 3H).

5-Azidopentanoic acid **(8):** To a solution of ethyl 5-azidopentanoate (3.64 g, 21.3 mmol) in THF and water (3:1, 24 mL) was added a solution of LiOH·H_2O (3.13 g, 74.6 mmol) in H_2O (15 mL). The reaction mixture was stirred at room temperature for 6 h. After reaction, the organic solution was concentrated under vacuum. The residue was dissolved in 100 mL of EtOAc and washed with 1 M HCl (2 × 50 mL), H_2O (3 × 50 mL) and brine (50 mL). The organic layer was dried over Na_2SO_4, filtered, and concentrated under vacuum to afford 5-azidopentanoic acid (2.56 g, 84% yield). **^{1}H NMR** (400 MHz, $CDCl_3$, δ): 3.30 (t, *J* = 6.8 Hz, 2H), 2.40 (t, *J* = 6.8 Hz, 2H), 1.78-1.57 (m, 4H).

Synthesis of 4-(azidomethyl)benzoic acid (azido molecules **9).**[S9]

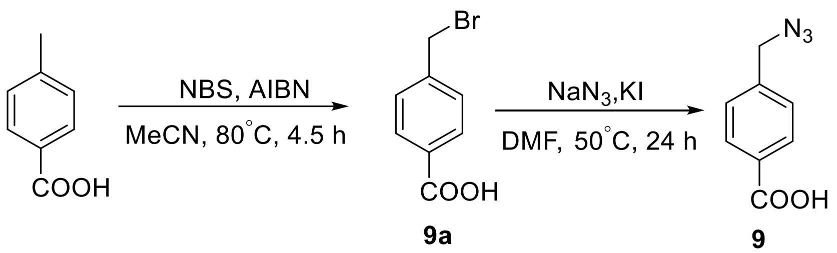

9a
9

4-(Bromomethyl)benzoic acid **(9a):** To a solution of *p*-toluic acid (5 g, 36.7 mmol) in 100 mL of acetonitrile was added portionwise of *N*-bromosuccinimide (6.86 g, 38.54 mmol) and AIBN (150.5 mg, 2

mol%). The reaction mixture was stirred at 80 °C for 4.5 h. After reaction, the mixture was cooled to room temperature. The crude product was filtered, washed with acetonitrile and further purified by recrystallised from minimal methanol to afford 4-(bromomethyl)benzoic acid (6.16 g, 78% yield). **^{1}H NMR** (400 MHz, DMSO-d_6, δ): 7.93 (d, *J* = 8.4 Hz, 2H), 7.56 (d, *J* = 8.4 Hz, 2H), 4.76 (s, 2H).

4-(Azidomethyl)benzoic acid **(9):** 4-(bromomethyl)benzoic acid (3.0 g, 14.0 mmol) and NaN_3 (1.82 g, 28.0 mmol) were added in 50 mL of anhydrous DMF. The reaction mixture was stirred at 50 °C for 24 h under N_2 atmosphere. After reaction, the organic solution was evaporated under vacuum. The residue was dissolved in 100 mL of H_2O followed by addition of 1 M HCl. The resulting participate was dissolved in 100 mL of DCM, dried over Na_2SO_4, filtered and concentrated under vacuum to afford 4-(azidomethyl)benzoic acid (2.03 g, 82% yield). **^{1}H NMR** (400 MHz, DMSO-d_6, δ): 7.96 (d, *J* = 7.6 Hz, 2H), 7.49 (d, *J* = 7.6 Hz, 2H), 4.56 (s, 2H).

The full ^{1}H NMR spectra of these azido molecules were shown in **Figure S10-30**.

2. Supporting Data

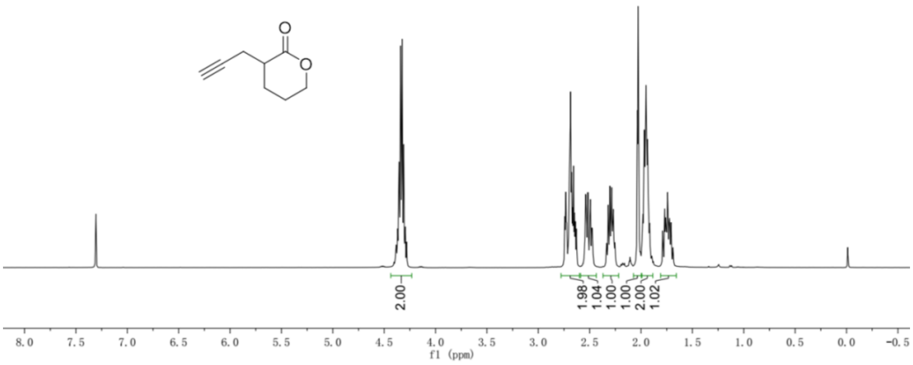

Figure S1. ^{1}H NMR spectra of AVL monomer.

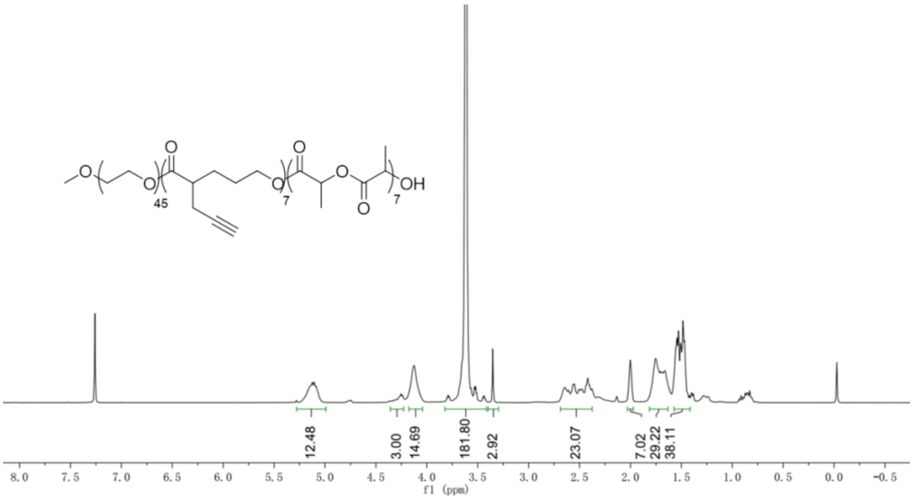

Figure S2. ^{1}H NMR spectra of mPEG$_{2K}$-*b*-P(AVL-*co*-LA)$_{2K}$ (AVL:LA =1:1) copolymer.

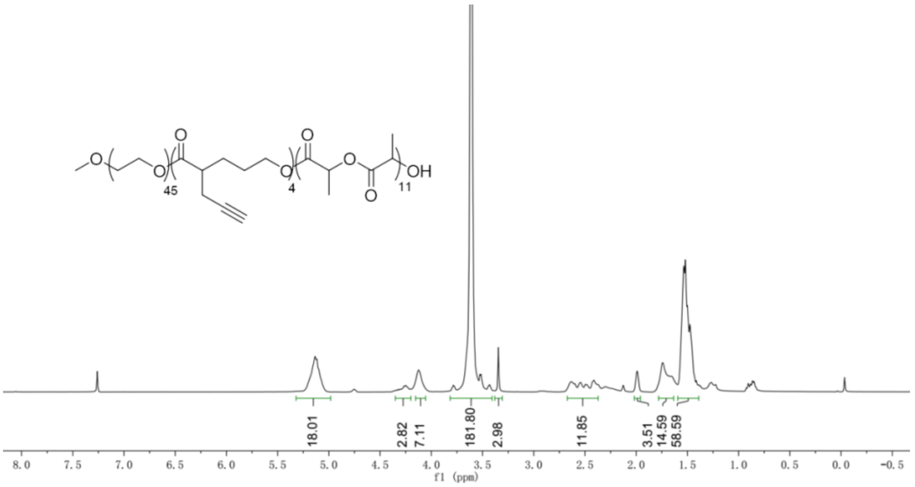

Figure S3. ^{1}H NMR spectra of mPEG$_{2K}$-*b*-P(AVL-*co*-LA)$_{2K}$ (AVL:LA =1:3) copolymer.

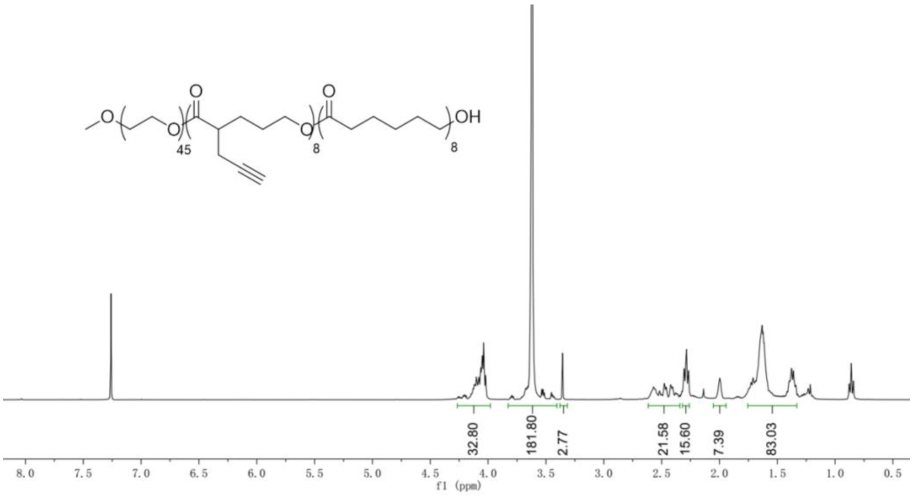

Figure S4. ^{1}H NMR spectra of mPEG$_{2K}$-*b*-P(AVL-*co*-CL)$_{2K}$ (AVL:CL=1:1) copolymer.

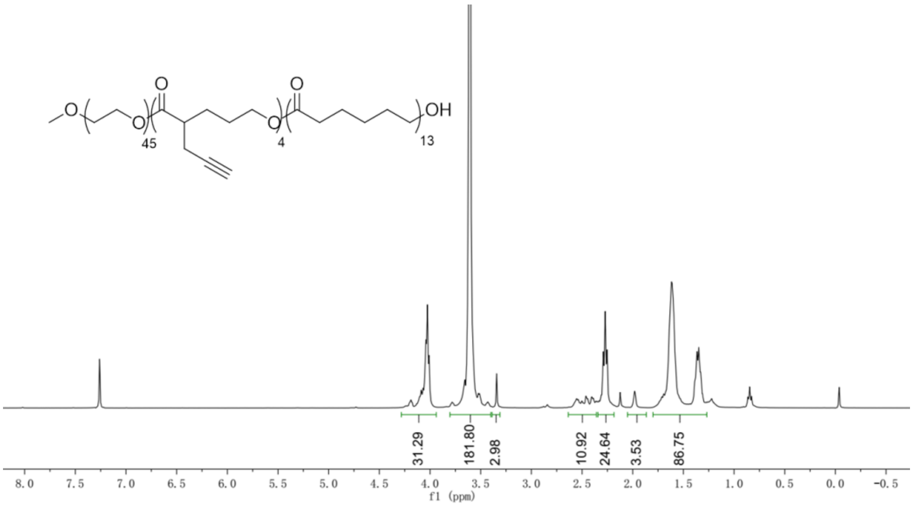

Figure S5. ^{1}H NMR spectra of mPEG$_{2K}$-*b*-P(AVL-*co*-CL)$_{2K}$ (AVL:CL=1:3) copolymer.

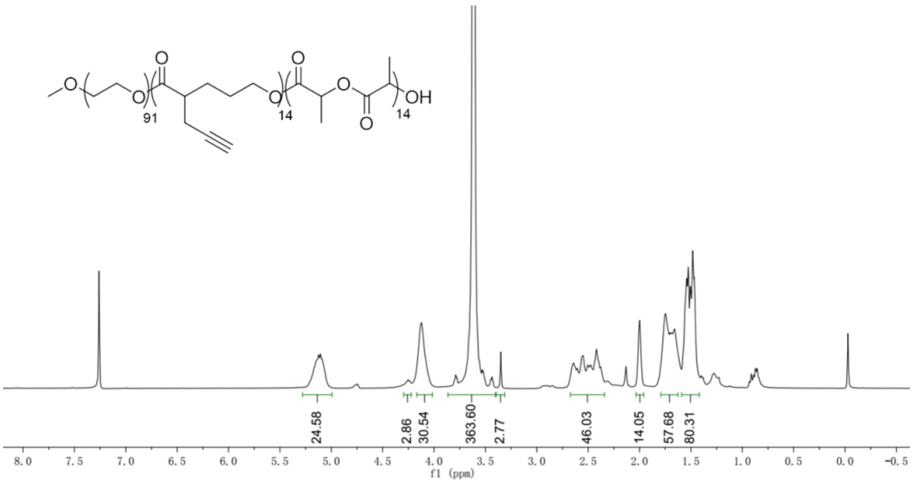

Figure S6. ^{1}H NMR spectra of mPEG$_{4K}$-*b*-P(AVL-*co*-LA)$_{4K}$ (AVL:LA =1:1) copolymer.

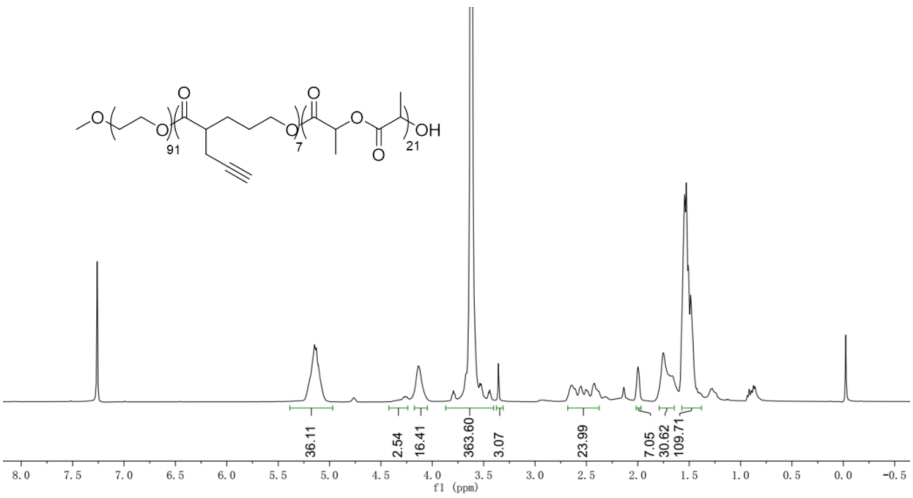

Figure S7. ^{1}H NMR spectra of mPEG$_{4K}$-*b*-P(AVL-*co*-LA)$_{4K}$ (AVL:LA =1:3) copolymer.

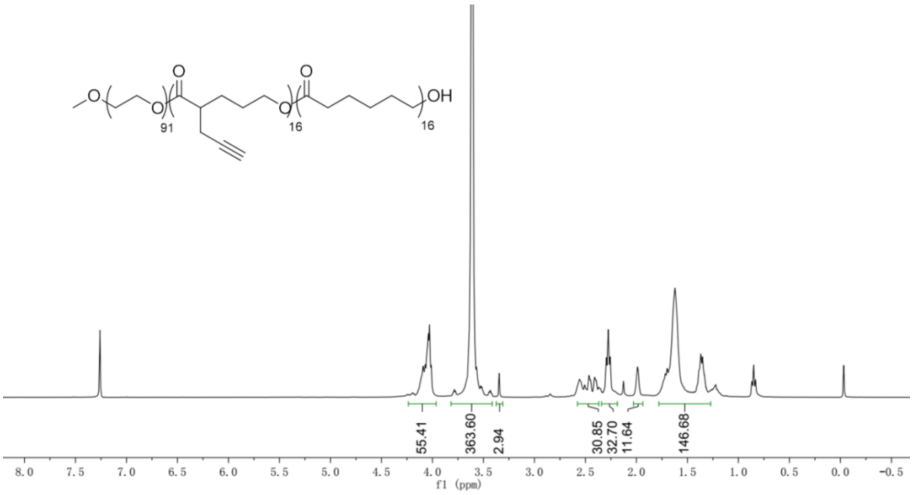

Figure S8. ^{1}H NMR spectra of mPEG$_{4K}$-*b*-P(AVL-*co*-CL)$_{4K}$ (AVL:CL=1:1) copolymer.

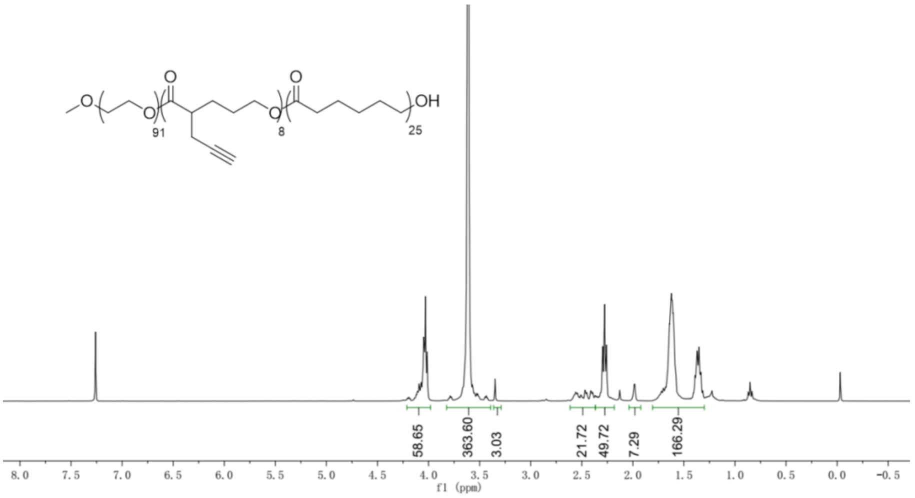

Figure S9. ^{1}H NMR spectra of mPEG$_{4K}$-*b*-P(AVL-*co*-CL)$_{4K}$ (AVL:CL=1:3) copolymer.

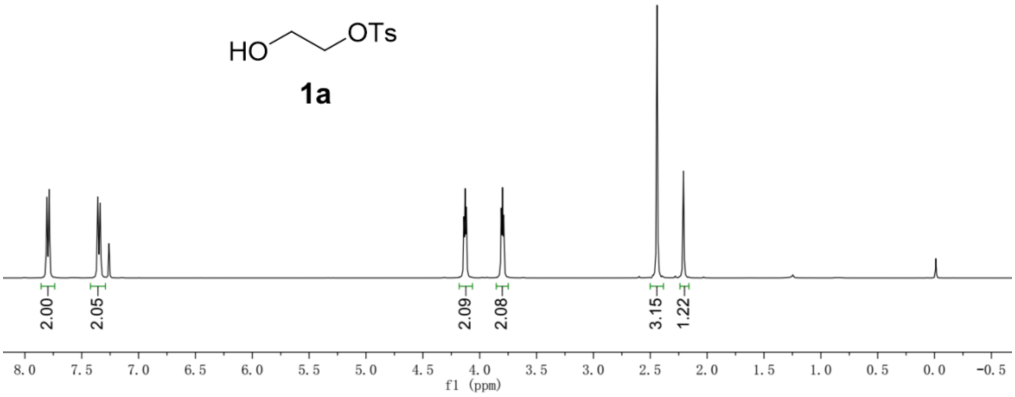

Figure S10. ^{1}H NMR spectra of 2-Hydroxyethyl-*p*-toluenesulfonate (**1a**).

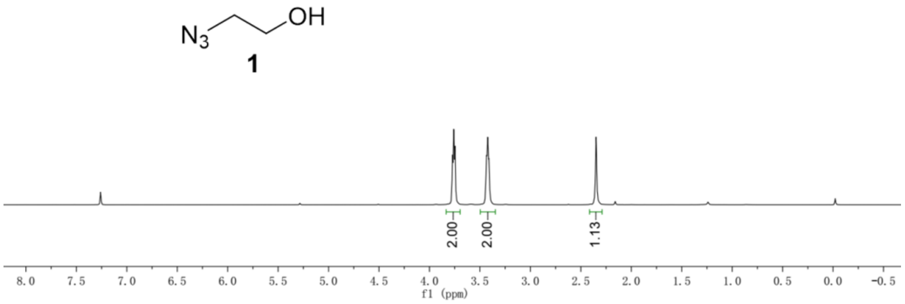

Figure S11. ^{1}H NMR spectra of 2-azidoethanol (**1**).

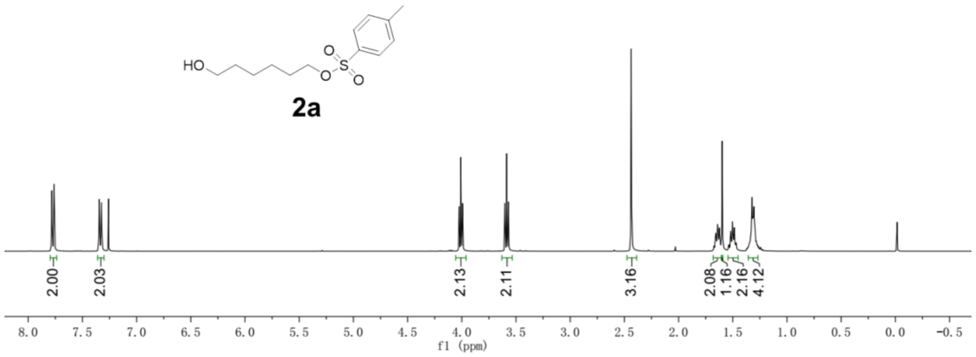

Figure S12. ^{1}H NMR spectra of 6-hydroxyethyl-*p*-toluenesulfonate (**2a**).

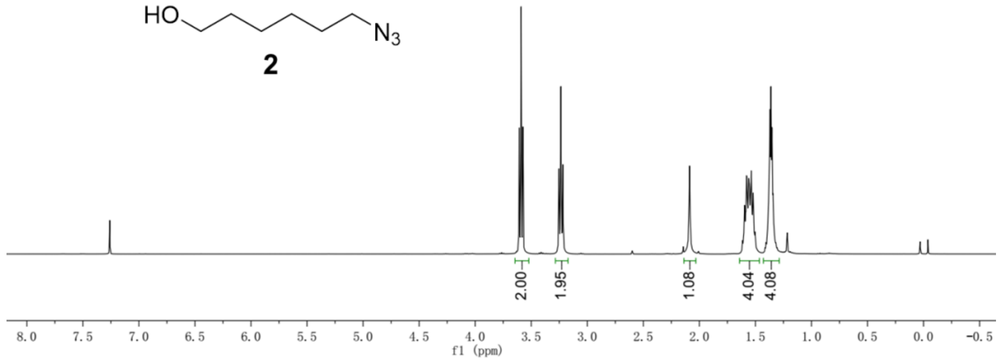

Figure S13. ^{1}H NMR spectra of 6-azidohexan-1-ol (**2**).

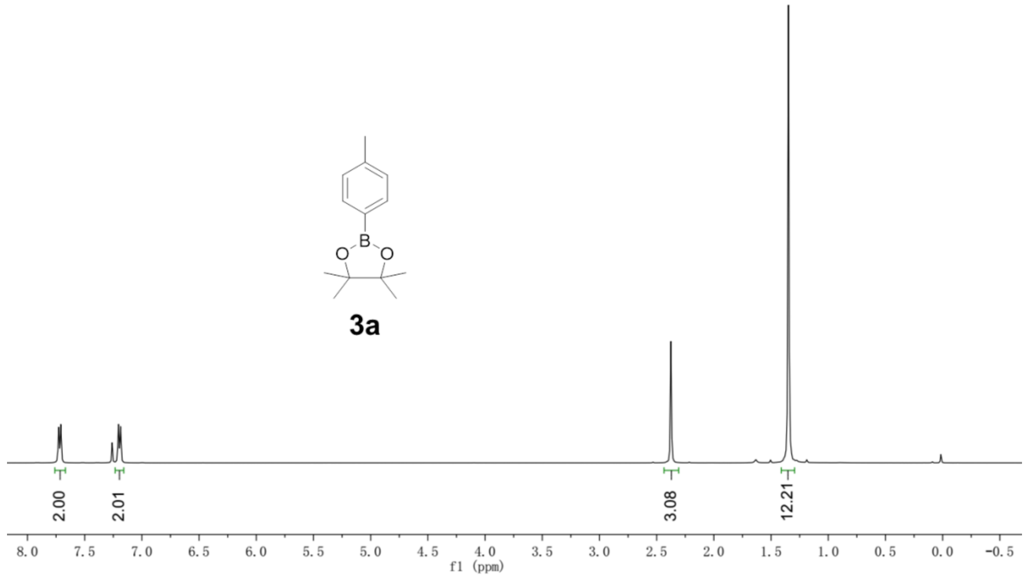

Figure S14. ^{1}H NMR spectra of 4,4,5,5-tetramethyl-2-(*p*-tolyl)-1,3,2-dioxaborolane (**3a**).

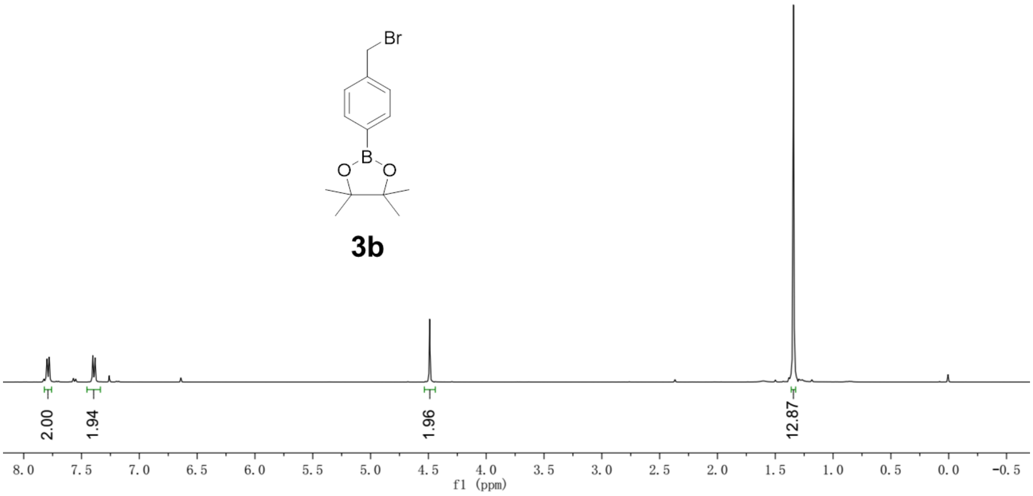

Figure S15. ^{1}H NMR spectra of 2-(4-(bromomethyl)phenyl)-4,4,5,5-tetramethyl-1,3,2-dioxaborolane (**3b**).

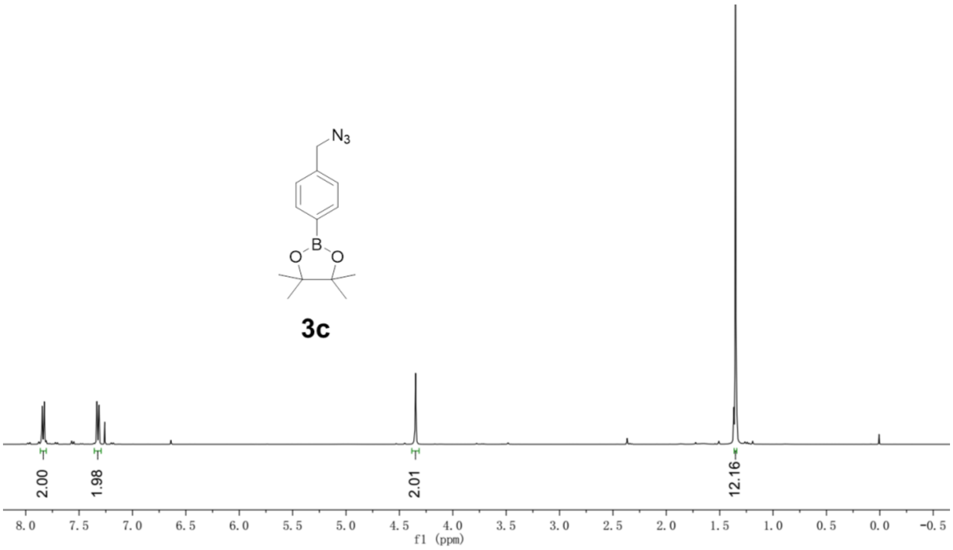

Figure S16. ^{1}H NMR spectra of 2-(4-(azidomethyl)phenyl)-4,4,5,5-tetramethyl-1,3,2-dioxaborolane (**3c**).

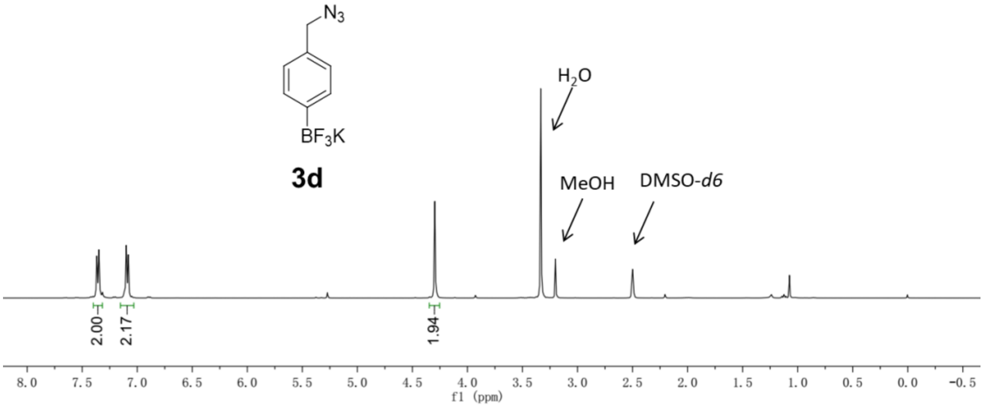

Figure S17. ^{1}H NMR spectra of potassium 4-(azidomethyl)phenyltrifluoroborate (**3d**).

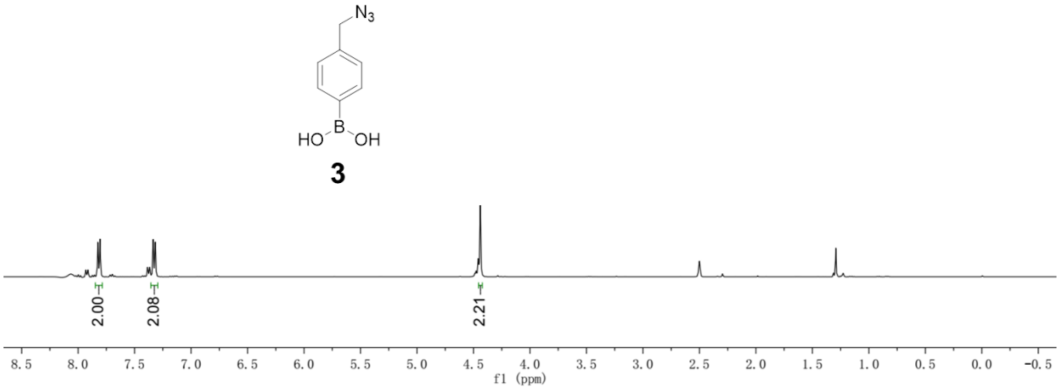

Figure S18. ^{1}H NMR spectra of [4-(azidomethyl)phenyl]boronic acid (**3**).

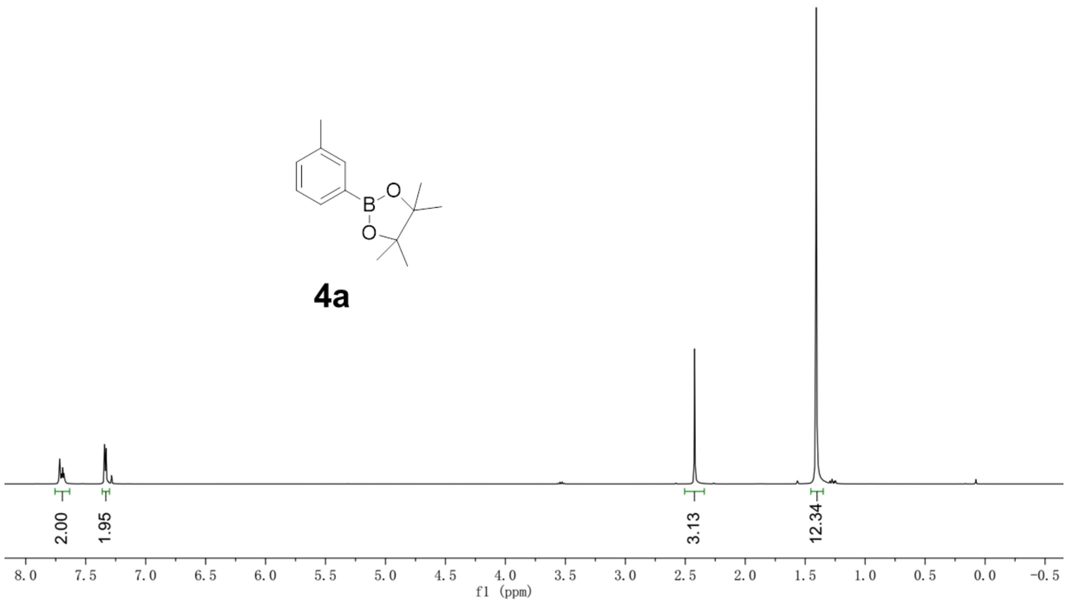

Figure S19. ^{1}H NMR spectra of 4,4,5,5-Tetramethyl-2-(*m*-tolyl)-1,3,2-dioxaborolane (**4a**).

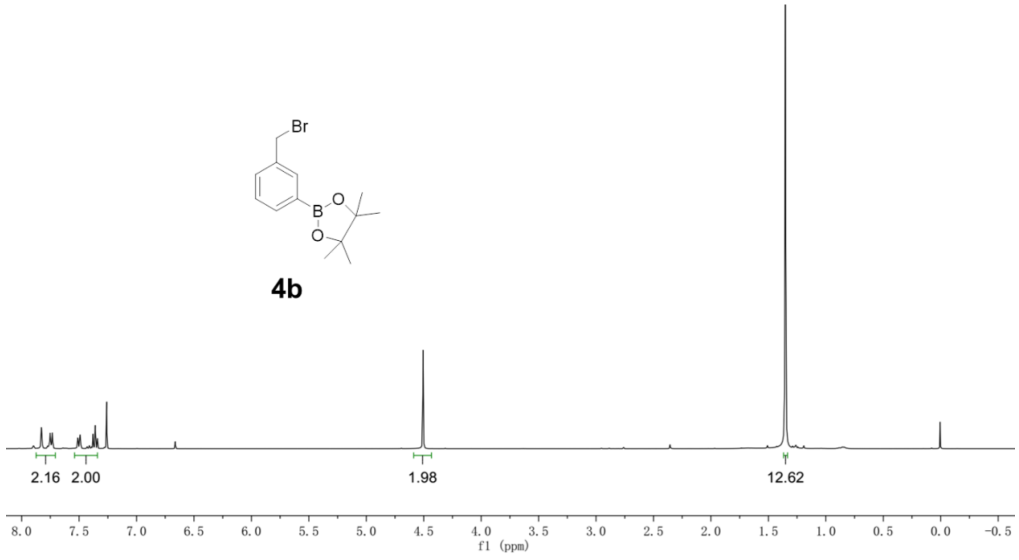

Figure S20. ^{1}H NMR spectra of 2-(3-(Bromomethyl)phenyl)-4,4,5,5-tetramethyl-1,3,2-dioxaborolane (**4b**).

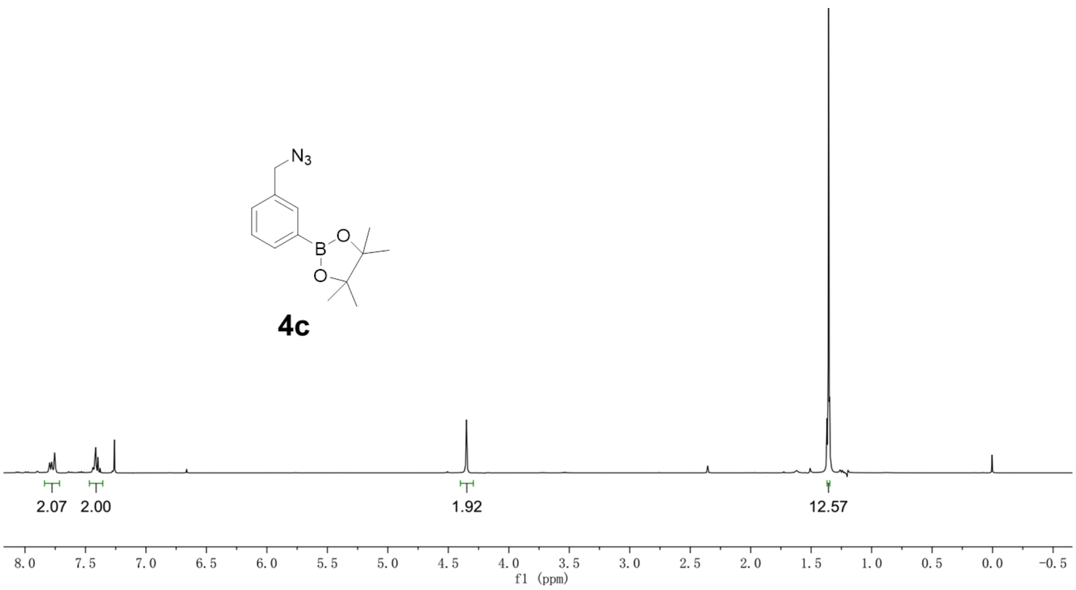

Figure S21. ^{1}H NMR spectra of 2-(3-(azidomethyl)phenyl)-4,4,5,5-tetramethyl-1,3,2-dioxaborolane (**4c**).

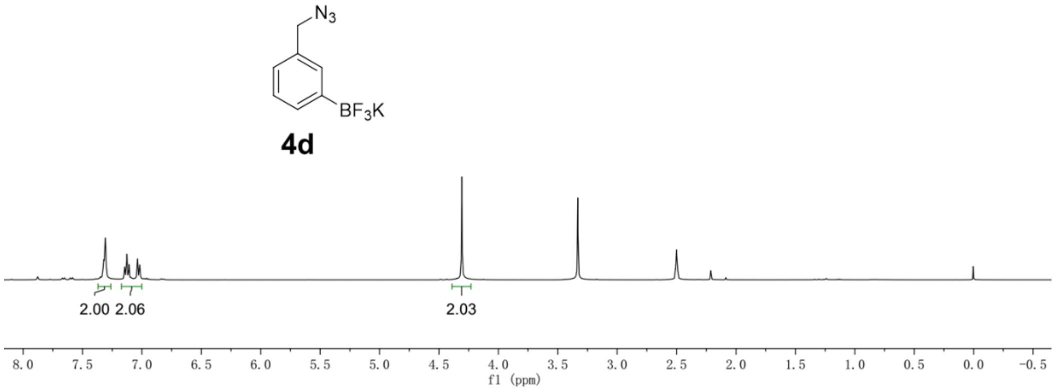

Figure S22. ^{1}H NMR spectra of potassium 3-(azidomethyl)phenyltrifluoroborate (**4d**).

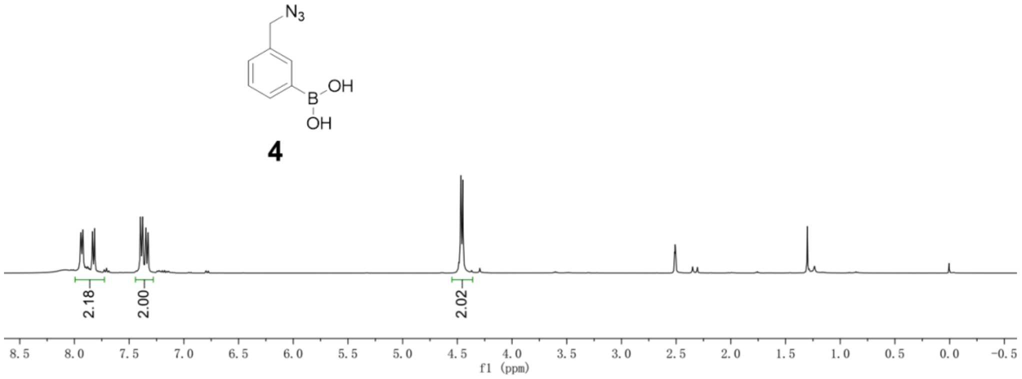

Figure S23. ^{1}H NMR spectra of potassium [3-(azidomethyl)phenyl]boronic acid (**4**).

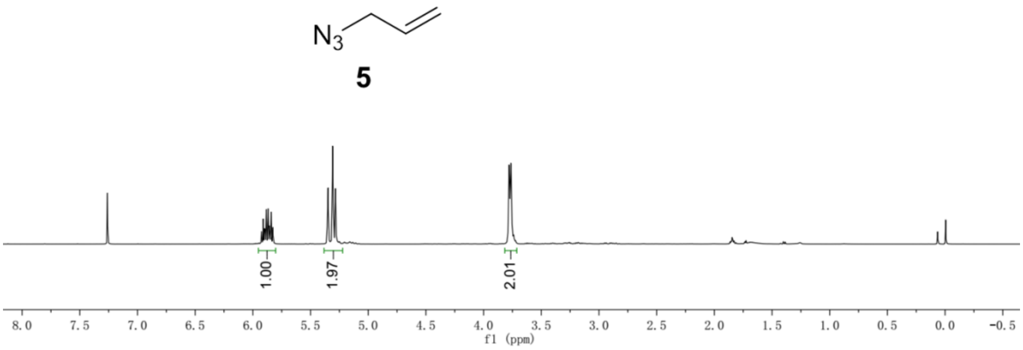

Figure S24. ^{1}H NMR spectra of 3-azidoprop-1-ene (**5**).

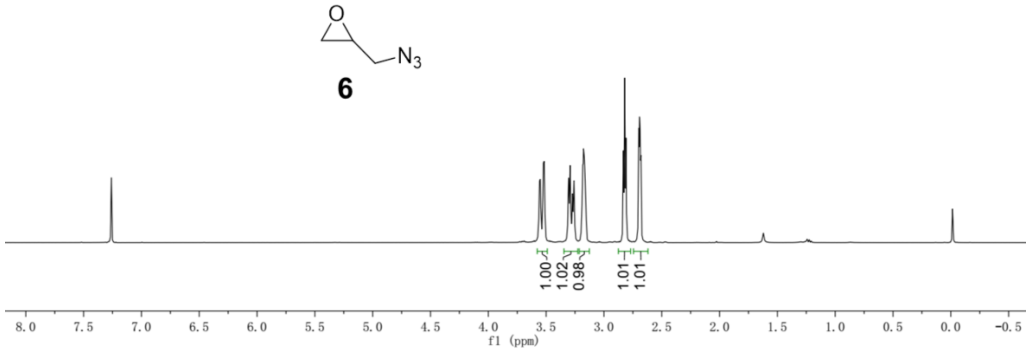

Figure S25. ^{1}H NMR spectra of 2-(azidomethyl)oxirane (**6**).

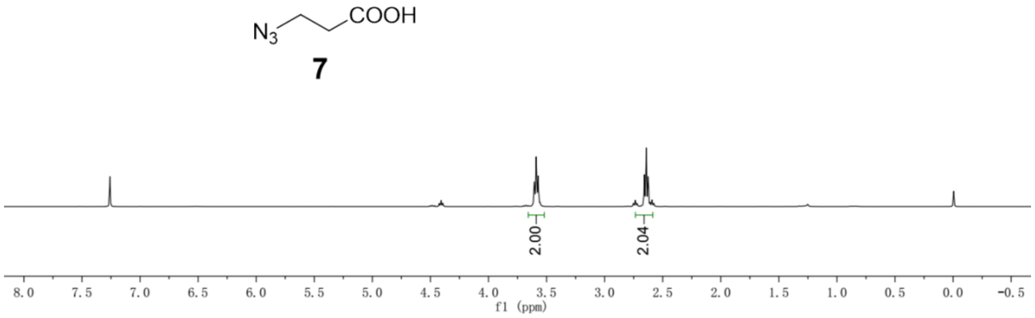

Figure S26. ^{1}H NMR spectra of 3-azidopropanoic acid (**7**).

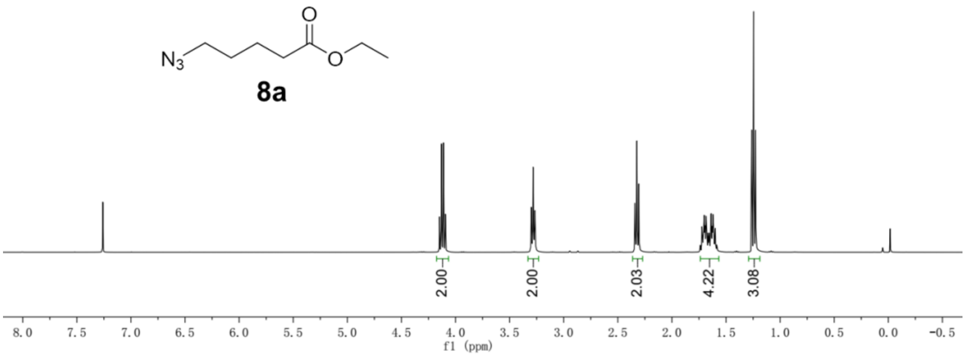

Figure S27. ^{1}H NMR spectra of ethyl 5-azidopentanoate (**8a**).

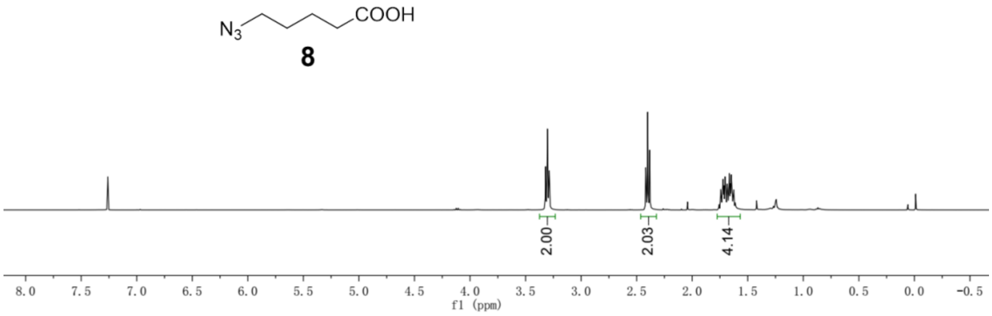

Figure S28. ^{1}H NMR spectra of 5-azidopentanoic acid (**8**).

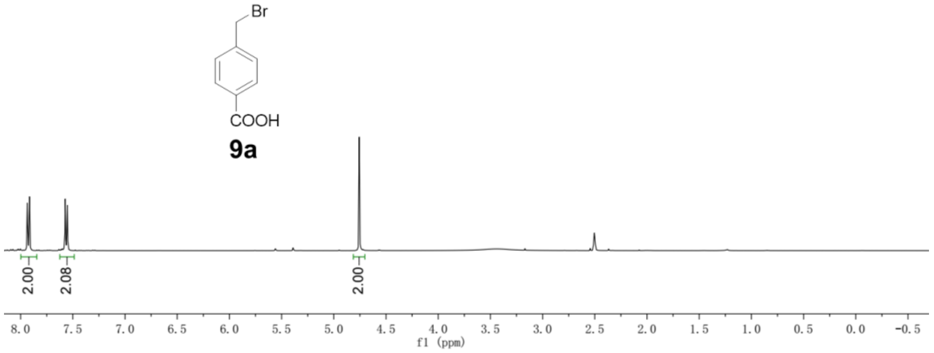

Figure S29. ^{1}H NMR spectra of 4-(bromomethyl)benzoic acid (**9a**).

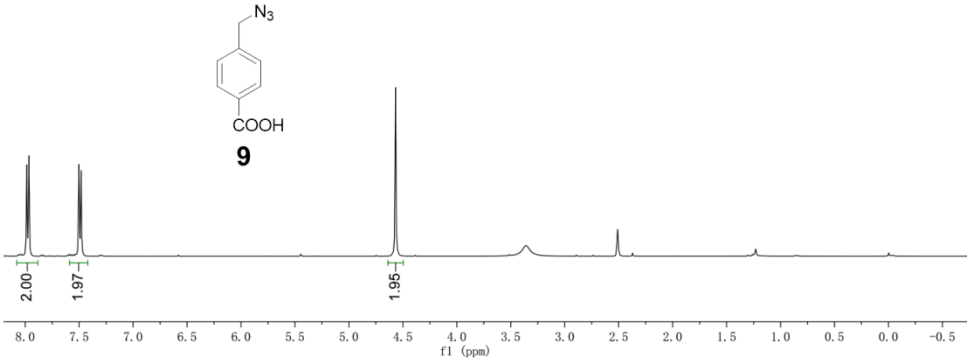

Figure S30. ^{1}H NMR spectra of 4-(azidomethyl)benzoic acid (**9**).

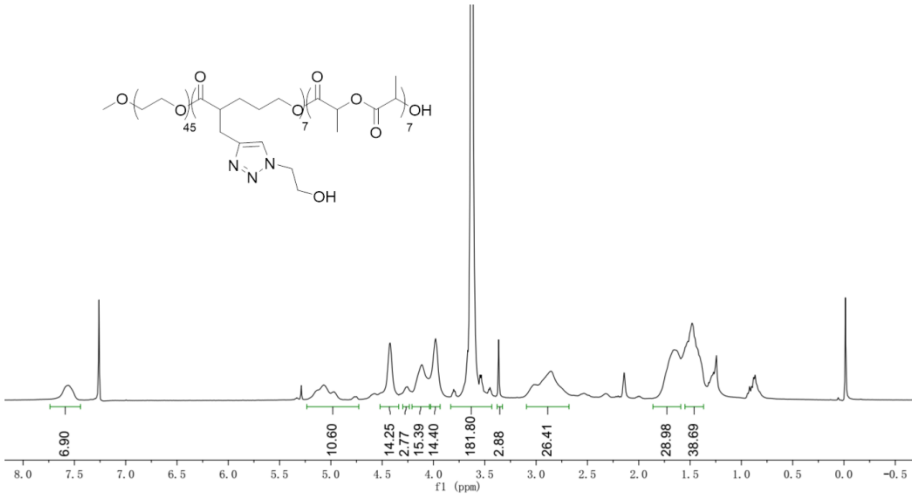

Figure S31. ^{1}H NMR spectra of **PPAL$_1$ 1** copolymer.

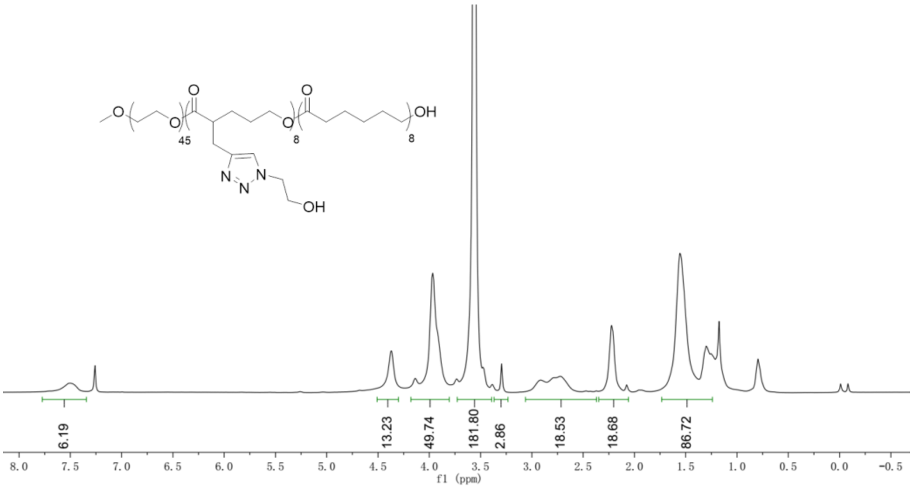

Figure S32. ^{1}H NMR spectra of **PPAC$_1$ 1** copolymer.

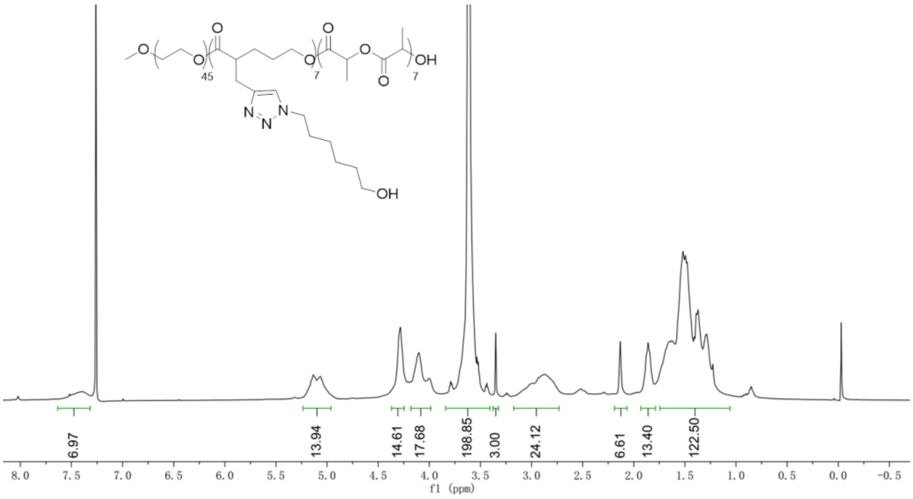

Figure S33. ^{1}H NMR spectra of **PPAL$_1$ 2** copolymer.

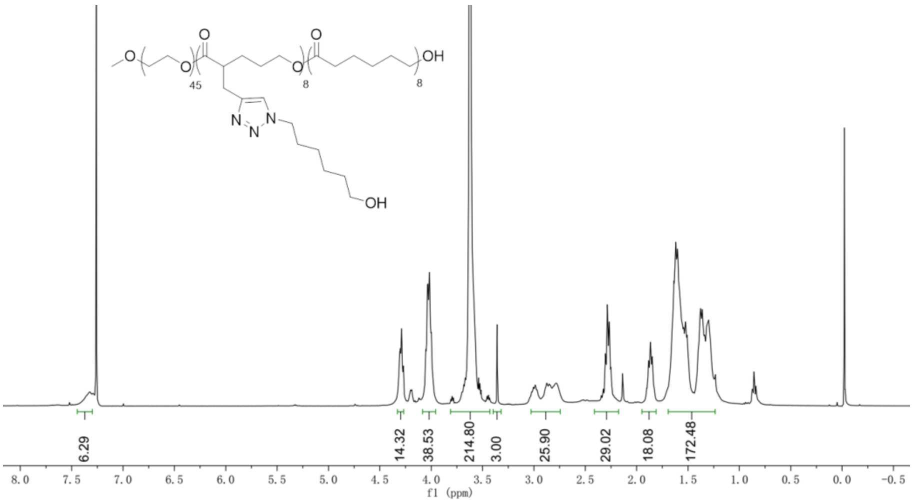

Figure S34. ^{1}H NMR spectra of **PPAC$_1$ 2** copolymer.

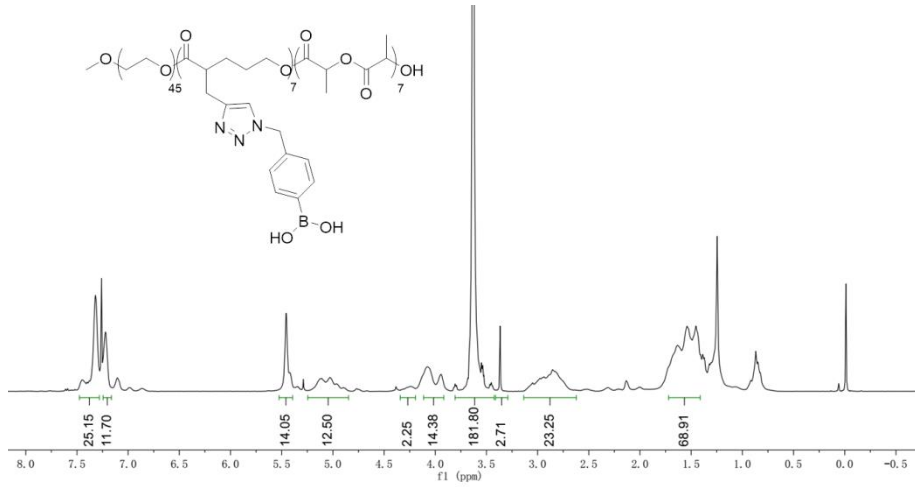

Figure S35. ^{1}H NMR spectra of **PPAL$_1$ 3** copolymer.

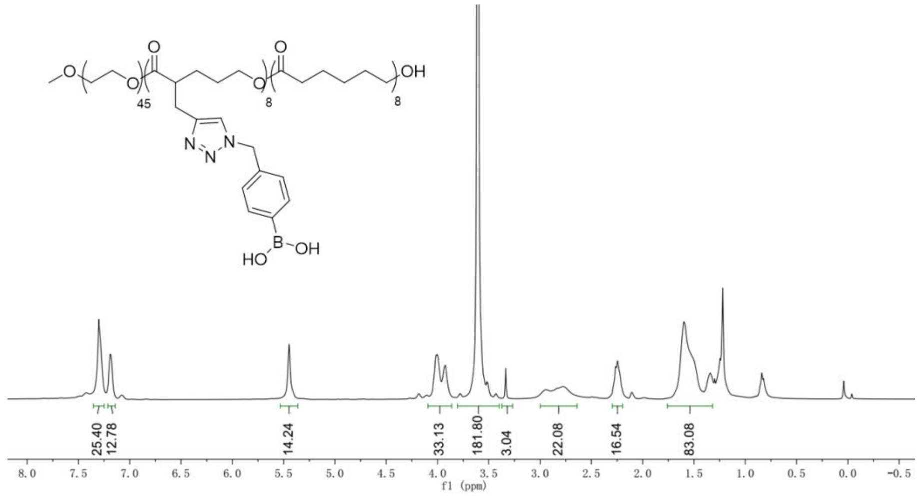

Figure S36. ^{1}H NMR spectra of **PPAC$_1$ 3** copolymer.

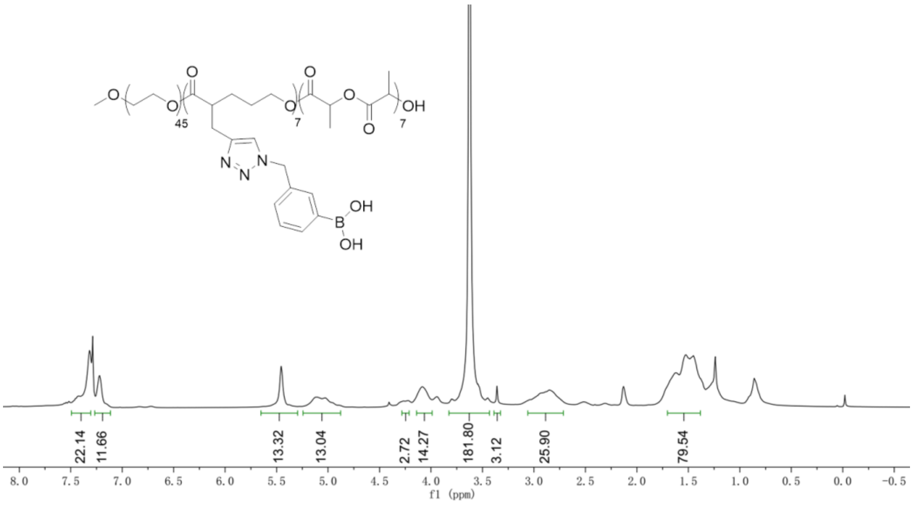

Figure S37. ^{1}H NMR spectra of **PPAL$_1$ 4** copolymer.

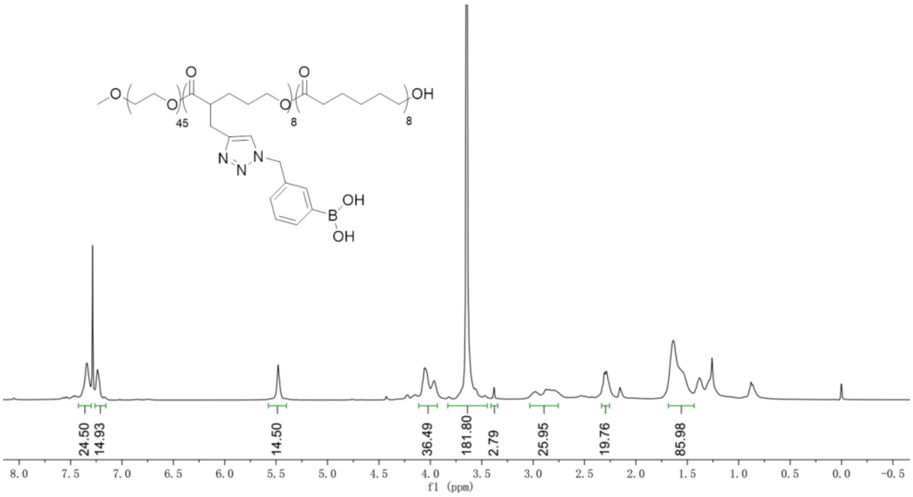

Figure S38. ^{1}H NMR spectra of **PPAC$_1$ 4** copolymer.

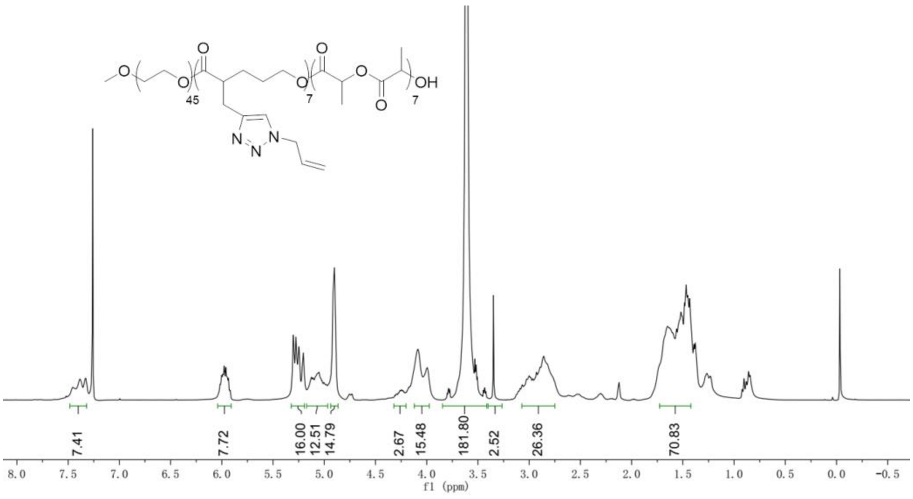

Figure S39. ^{1}H NMR spectra of **PPAL$_1$ 5** copolymer.

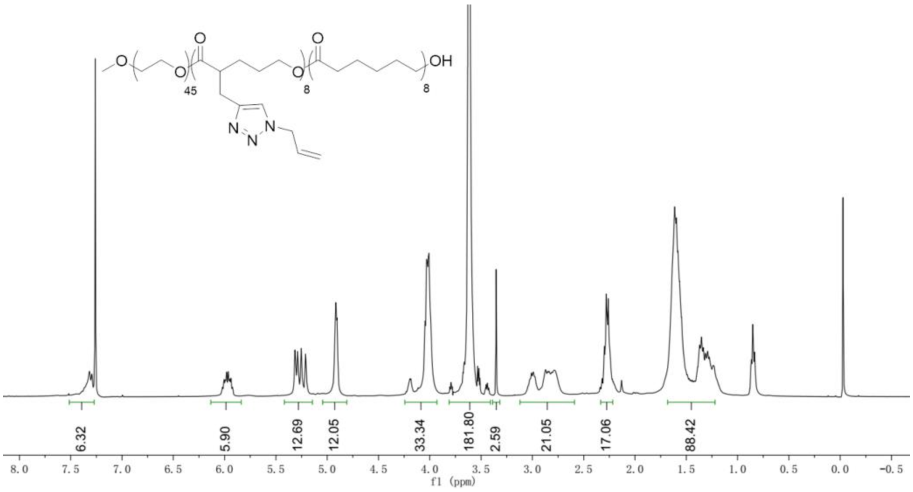

Figure S40. ^{1}H NMR spectra of **PPAC$_1$ 5** copolymer.

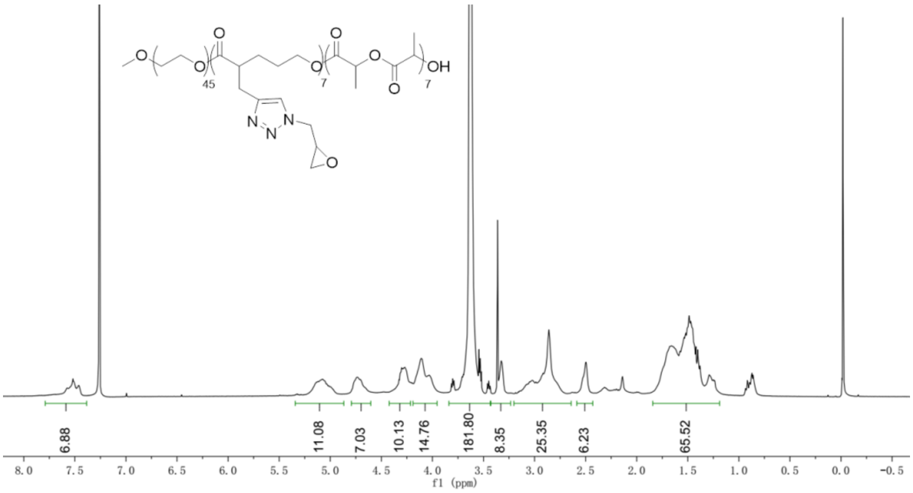

Figure S41. ^{1}H NMR spectra of **PPAL$_1$ 6** copolymer.

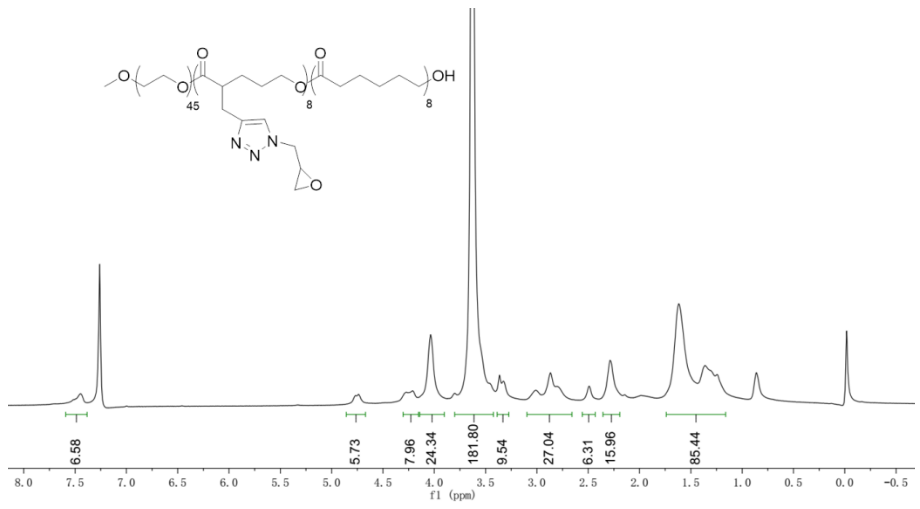

Figure S42. ^{1}H NMR spectra of **PPAC$_1$ 6** copolymer.

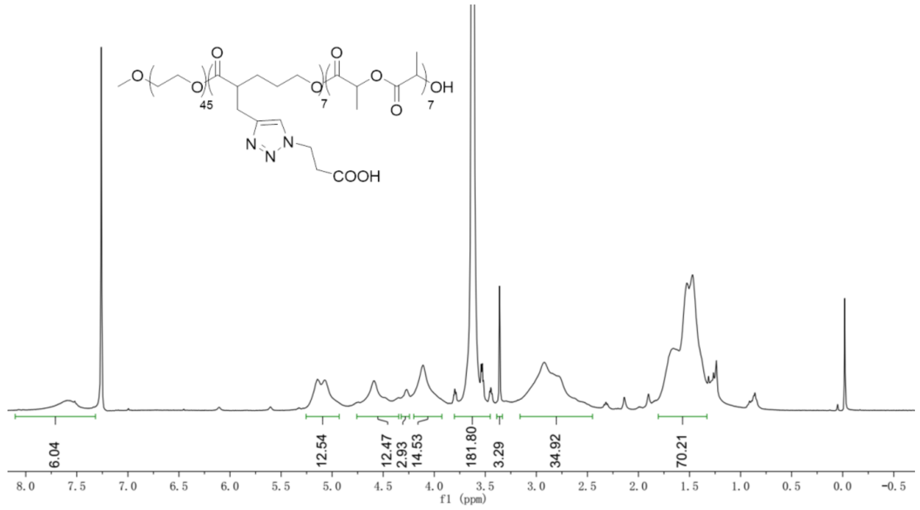

Figure S43. ^{1}H NMR spectra of **PPAL$_1$ 7** copolymer.

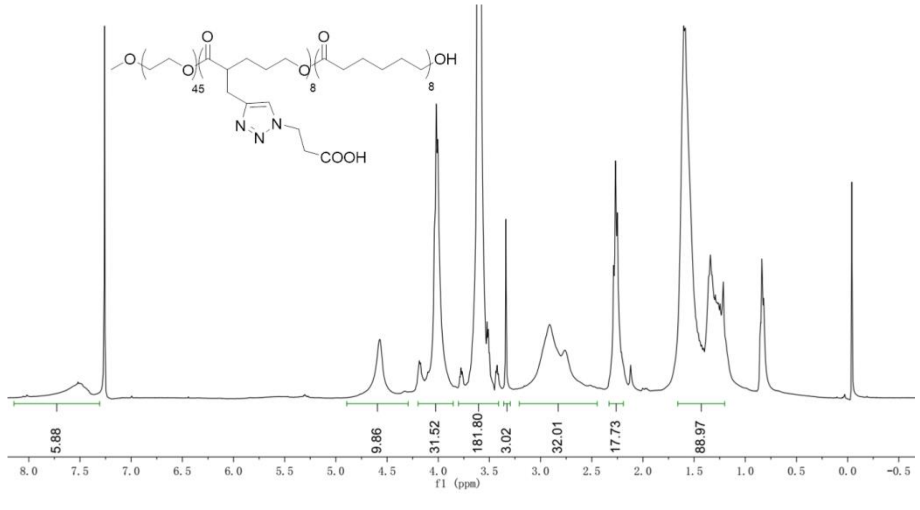

Figure S44. ^{1}H NMR spectra of **PPAC$_1$ 7** copolymer.

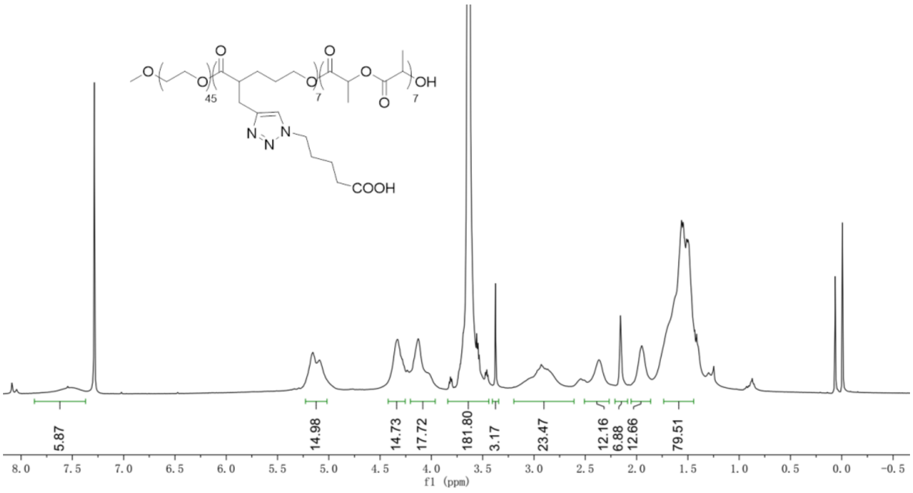

Figure S45. ^{1}H NMR spectra of **PPAL$_1$ 8** copolymer.

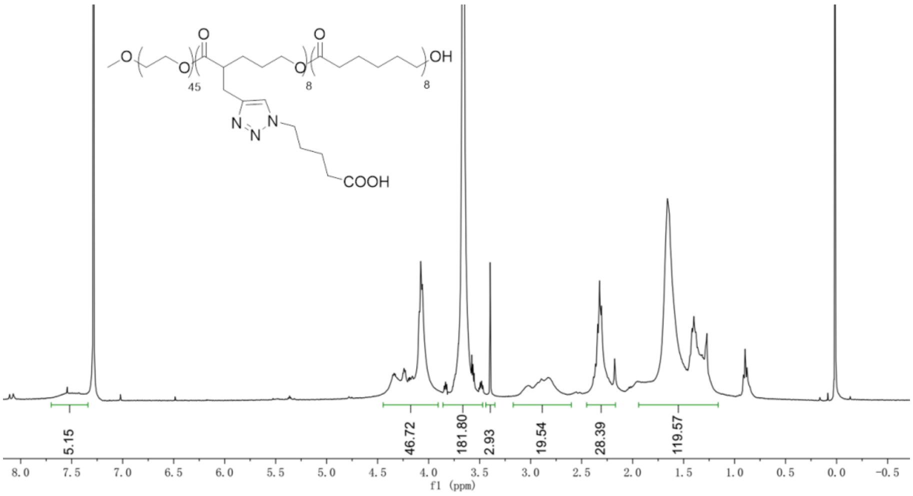

Figure S46. ^{1}H NMR spectra of **PPAC$_1$ 8** copolymer.

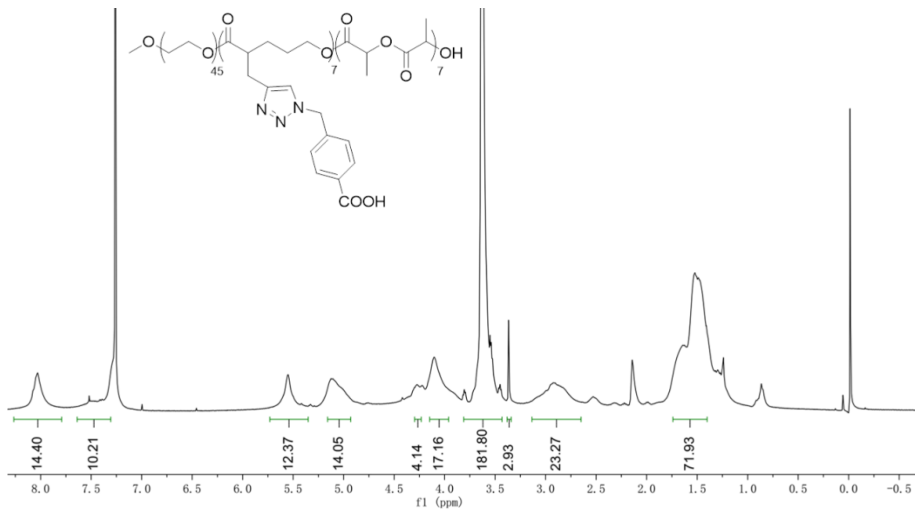

Figure S47. ^{1}H NMR spectra of **PPAL$_1$ 9** copolymer.

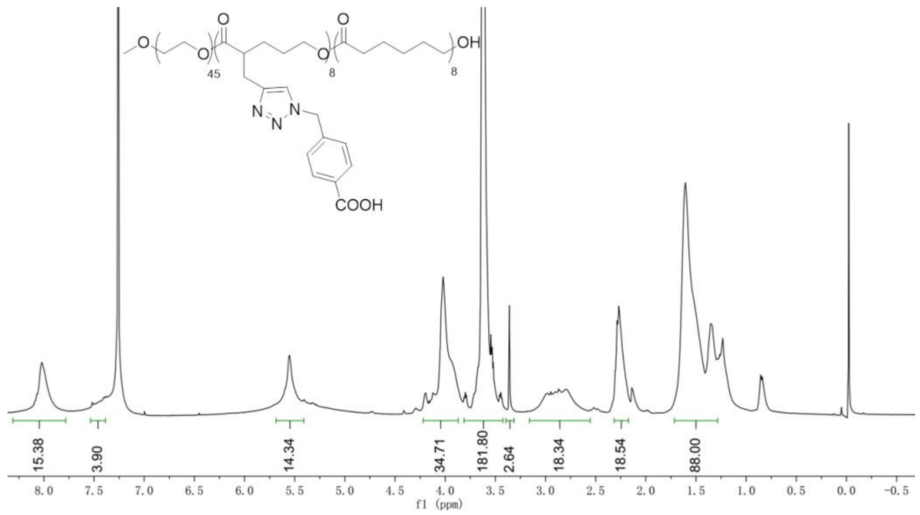

Figure S48. ^{1}H NMR spectra of **PPAC$_1$ 9** copolymer.

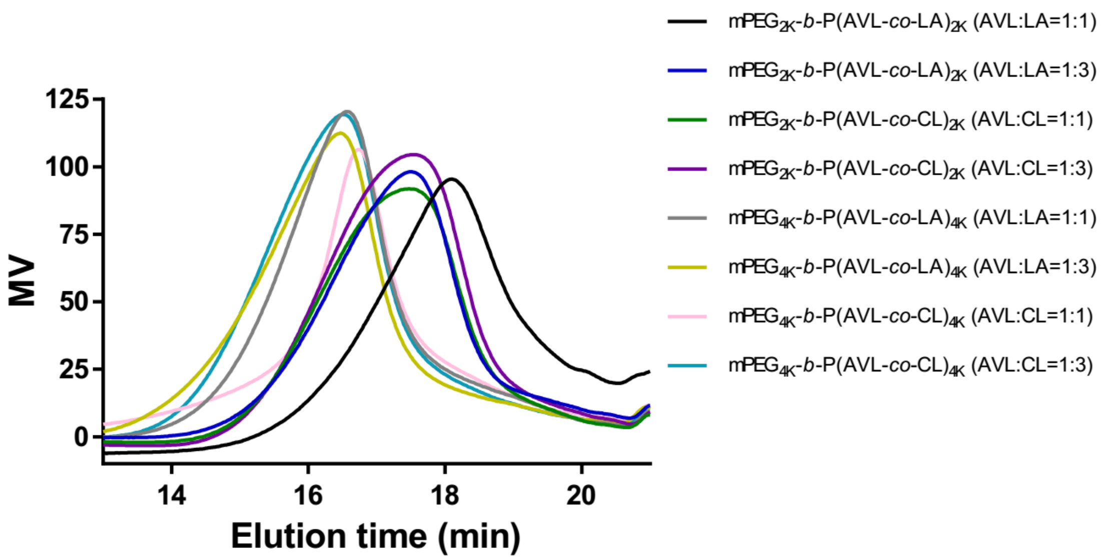

Figure S49. GPC curve of **PPAL** and **PPAC** copolymers in THF at 30°C with 1 mL/min flow rate.

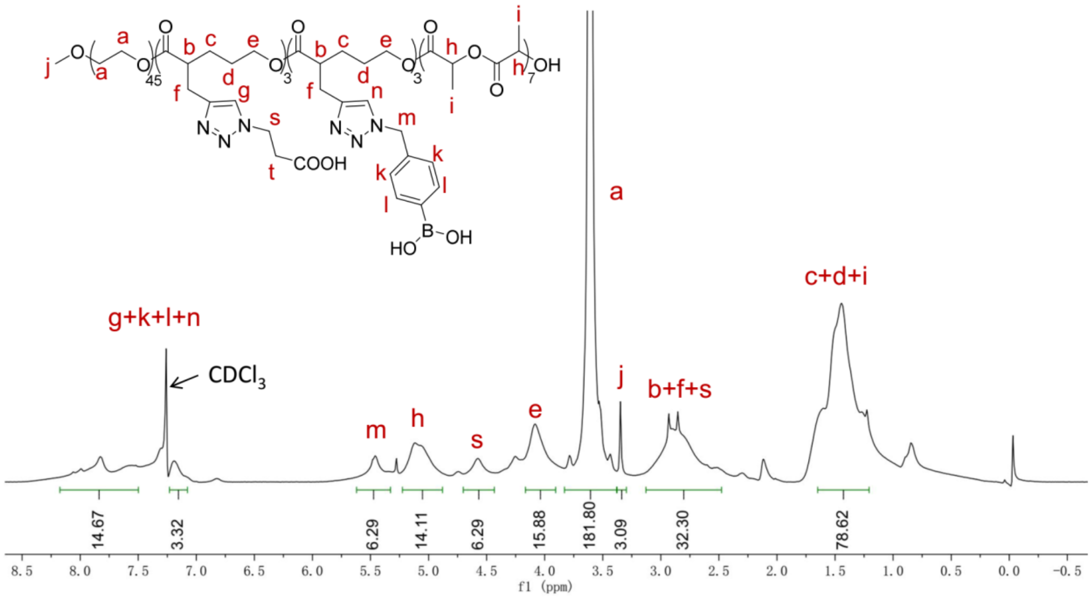

Figure S50. ^{1}H NMR spectra of $\mathbf{PPAL_1}$ **3, 7** copolymer.

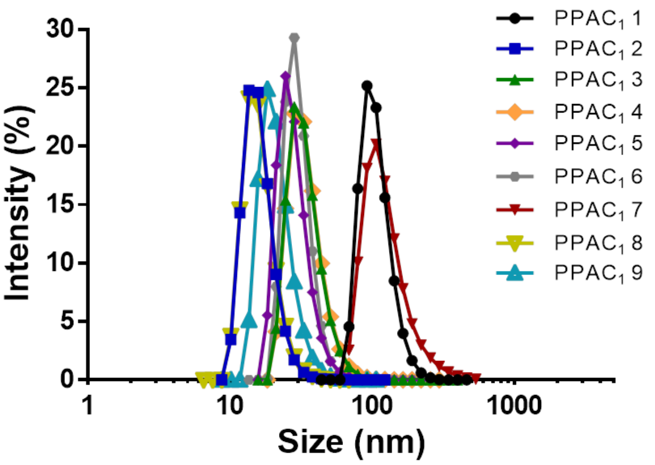

Figure S51. Dynamic light scattering curves of **PPAC$_1$ 1-9** nanoparticles in aqueous media.

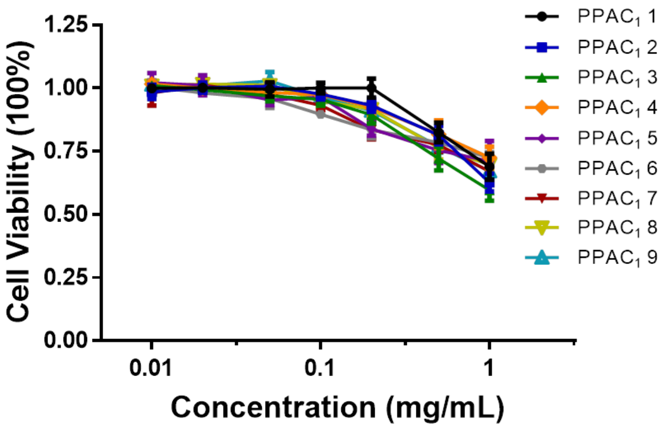

Figure S52. *In vitro* cytotoxicity of **PPAC$_1$ 1-9** copolymers in HeLa cells at various concentrations.

Table S1. Results of **PPAC$_1$** copolymers click with azido molecules via CuAAC reaction.

	mPEG$_{2K}$-*b*-P(AVL-*co*-CL)$_{2K}$ (AVL:CL=1:1) (PPAC$_1$)		
**Azido molecule**	**Grafting efficiency (%)**	**Yield (%)**	**$M_{n,NMR}$ (g/mol)**
**1**	94.5	71.5	4500
**2**	99.3	80.0	4800

**3**	100	82.7	5100
**4**	91.8	80.2	5300
**5**	85.5	77.0	5000
**6**	89.0	89.5	4700
**7**	79.5	75	4600
**8**	83.8	80.0	5000
**9**	99.3	67.8	4900

Table S2. Functionalization of **PPAL** and **PPAC** copolymers with azido molecules **3** via CuAAC reaction.

	**Azido molecule 3**	
**Copolymer**	**Grafting efficiency (%)**	**Yield (%)**
$mPEG_{2K}$-*b*-P(AVL-*co*-LA)$_{2K}$ (AVL:LA=1:1) **($PPAL_1$)**	100.0	82.5
$mPEG_{2K}$-*b*-P(AVL-*co*-LA)$_{2K}$ (AVL:LA =1:3) **($PPAL_2$)**	100.0	93.0
$mPEG_{4K}$-*b*-P(AVL-*co*-LA)$_{4K}$ (AVL:LA=1:1) **($PPAL_3$)**	93.9	76.6
$mPEG_{4K}$-*b*-P(AVL-*co*-LA)$_{4K}$ (AVL:LA=1:3) **($PPAL_4$)**	100.0	78.0
$mPEG_{2K}$-*b*-P(AVL-*co*-CL)$_{2K}$ (AVL:CL=1:1) **($PPAC_1$)**	100.0	82.7
$mPEG_{2K}$-*b*-P(AVL-*co*-CL)$_{2K}$ (AVL:CL=1:3) **($PPAC_2$)**	74.4	82.0
$mPEG_{4K}$-*b*-P(AVL-*co*-CL)$_{4K}$ (AVL:CL=1:1) **($PPAC_3$)**	95.7	90.5
$mPEG_{4K}$-*b*-P(AVL-*co*-CL)$_{4K}$ (AVL:CL=1:3) **($PPAC_4$)**	70.8	84.6

Table S3. Characterization of **$PPAC_1$ 1-9** copolymer nanoparticles.

**Functional copolymer**	**Particle size (nm)**	**PDI**
$PPAC_1$ 1	135.52 ± 3.2	0.153 ± 0.02
$PPAC_1$ 2	20.56 ± 3.5	0.282 ± 0.04

$PPAC_1$ 3	30.56 ± 1.2	0.140 ± 0.02
$PPAC_1$ 4	29.25 ± 3.5	0.259 ± 0.01
$PPAC_1$ 5	28.44 ± 1.2	0.201 ± 0.04
$PPAC_1$ 6	29.56 ± 2.4	0.248 ± 0.05
$PPAC_1$ 7	137.7 ± 11.05	0.141 ± 0.03
$PPAC_1$ 8	22.11 ± 2.6	0.218 ± 0.01
$PPAC_1$ 9	26.14 ± 3.5	0.261 ± 0.02

3. References

(1) M. Popr, S. Hybelbauerová, J. Jindřich, *Beilstein J. Org. Chem.* **2014,** *10,* 1390–1396.

(2) J. He, C. Fang, R. A. Shelp, M. B. Zimmt, *Langmuir* **2017,** *33,* 459–467.

(3) W. L. Zhai, B. M. Chapin, A. Yoshizawa, H.-C. Wang, S. A. Hodge, T. D. James, E. V. Anslyn, J. S. Fossey, *Org. Chem. Front.* **2016,** *3,* 918-928.

(4) A. K. L. Yuena, C. A. Hutton, *Tetrahedron Lett.* **2005,** *46,* 7899–7903.

(5) M. Swetha, P. V. Ramana, S. G. Shirodkar, *Org. Prep. Proced. Int.* **2011,** *43,* 348–353.

(6) E. G. Gharakhanian, T. J. Deming, *Biomacromolecules* **2015,** *16,* 1802–1806**.**

(7) P. E. Schneggenburger, A. Beerlink, B. Worbs, T. Salditt, U. Diederichsen, *ChemPhysChem* **2009,** *10,* 1567-1576.

(8) H. Kakwere, R. J. Payne, K. A. Jolliffe, S. Perrier, *Soft Matter* **2011,** *7,* 3754-3757.

(9) M. Wrobel, J. Aubé, B. König, *Beilstein J. Org. Chem.* **2012,** *8,* 1027–1036.
